# Molecular dissection of connected rice populations revealed important genomic regions for agronomic and biofortification traits

**DOI:** 10.3389/fpls.2023.1157507

**Published:** 2023-03-22

**Authors:** Alvin D. Palanog, Chau Thanh Nha, Gwen Iris L. Descalsota-Empleo, Mark Ian Calayugan, Zin Mar Swe, Amery Amparado, Mary Ann Inabangan-Asilo, Jose E. Hernandez, Pompe C. Sta. Cruz, Teresita H. Borromeo, Antonio G. Lalusin, Ramil Mauleon, Kenneth L. McNally, B. P. Mallikarjuna Swamy

**Affiliations:** ^1^ Rice Breeding Innovations Platform, International Rice Research Institute, Los Baños, Laguna, Philippines; ^2^ College of Agriculture and Food Science, University of the Philippines, Los Baños, Laguna, Philippines; ^3^ PhilRice Negros Branch Station, Philippine Rice Research Institute, Murcia, Negros Occidental, Philippines; ^4^ Cant Ho University, Cant Ho, Vietnam; ^5^ College of Agriculture, University of Southern Mindanao, Kabacan, North Cotabato, Philippines

**Keywords:** rice, biofortification, micronutrients, Zn, QTL, candidate genes, connected populations

## Abstract

Breeding staple crops with increased micronutrient concentration is a sustainable approach to address micronutrient malnutrition. We carried out Multi-Cross QTL analysis and Inclusive Composite Interval Mapping for 11 agronomic, yield and biofortification traits using four connected RILs populations of rice. Overall, MC-156 QTLs were detected for agronomic (115) and biofortification (41) traits, which were higher in number but smaller in effects compared to single population analysis. The MC-QTL analysis was able to detect important QTLs viz: *qZn_5.2_, qFe_7.1_, qGY_10.1_, qDF_7.1_, qPH_1.1_, qNT_4.1_, qPT_4.1_, qPL_1.2_, qTGW_5.1_, qGL_3.1_*, and *qGW_6.1_*, which can be used in rice genomics assisted breeding. A major QTL (*qZn_5.2_*) for grain Zn concentration has been detected on chromosome 5 that accounted for 13% of R^2^. In all, 26 QTL clusters were identified on different chromosomes. *qPH_6.1_* epistatically interacted with *qZn_5.1_* and *qGY_6.2_*. Most of QTLs were co-located with functionally related candidate genes indicating the accuracy of QTL mapping. The genomic region of *qZn_5.2_* was co-located with putative genes such as *OsZIP5*, *OsZIP9*, and *LOC_OS05G40490* that are involved in Zn uptake. These genes included polymorphic functional SNPs, and their promoter regions were enriched with *cis*-regulatory elements involved in plant growth and development, and biotic and abiotic stress tolerance. Major effect QTL identified for biofortification and agronomic traits can be utilized in breeding for Zn biofortified rice varieties.

## Introduction

Globally, more than 3 billion people depend on rice for their daily caloric intake and nutritional needs (Yadaw et al., 2006; Elert, 2014; Bouis and Saltzman, 2017). However, milled rice is a poor source of micronutrients; hence, majority of the resource-poor rice consumers without access to adequate nutrition suffer mineral deficiencies (Dipti et al., 2012; Goudia and Hash, 2015; Garcia-Oliveira et al., 2018; Goloran et al., 2019; Ludwig and Slamet-Loedin, 2019). Iron (Fe) and Zinc (Zn) malnutrition is common across all age groups, especially among children and women in the developing world (Bouis et al., 2002; Ahmed et al., 2016; Palanog et al., 2019; Gupta et al., 2020; Calayugan et al., 2021). Increasing the mineral density in the edible portion of the staple crops is a food-based approach to tackle mineral deficiencies. It is reported to be the most economical and sustainable solution to address malnutrition (Dipti et al., 2012; Bouis and Saltzman, 2017; Goloran et al., 2019; Ludwig and Slamet-Loedin, 2019). However, for the successful adoption and consumption of biofortified rice varieties, they should be high-yielding, agronomically-superior, and desirable in terms of grain quality (Swamy et al., 2016; Swamy et al., 2021b).

Rice has a vast amount of genetic diversity available in cultivars, landraces, and wild relatives (Khush, 1997; Jackson and Lettington, 2003; Huggins et al., 2019). In particular, *aus* is a genetically-distinct group of rice accessions mainly originated from Bangladesh and India (Garis et al., 2005; Norton et al., 2018; Swamy et al., 2018). They are well-known for their wider adaptability, tolerance to biotic and abiotic stresses, and nutritional value (Xu et al., 2006; Ali et al., 2011; Henry et al., 2011; Gamuyao et al., 2012; Zhu et al., 2016). Currently, *aus* accessions are being explored for Zn biofortification due to their high grain Zn concentration (Swamy et al., 2018; Rakotondramana et al., 2022).

Quantitative trait loci (QTL) mapping using bi-parental populations is widely popular in rice (Al-Shugeairy et al., 2014; Travis et al., 2015). However, it has limited recombination and lesser allelic diversity, which results in larger confidence intervals of the QTLs, and captures only a portion of the total genetic variation (Xu et al., 2016; Verdeprado et al., 2018). Further, QTLs have to be fine-mapped and validated in multiple genetic backgrounds for their use in Marker Assisted Breeding (MAB). An alternative approach is QTL mapping in multi-parent populations that can increase the power of detecting major QTLs, and helps to understand QTL interactions and QTL by genetic background effects (Wang et al., 2014; Descalsota et al., 2018). Multi-parent advanced generation intercross (MAGIC) and nested-association mapping (NAM) populations increase the probability of identifying precise QTL (Bandillo et al., 2013).

In this study, we developed four connected recombinant inbred lines (RILs) populations derived from *Kaliboro* (IRGC 77201-1) - a high grain Zn germplasm crossed with four elite Zn breeding lines. These populations were analyzed using multi-cross QTL (MC-QTL) method to identify QTLs for agronomic, yield and biofortification traits, candidate genes were shortlisted for major effect QTLs, and detailed analyses of major effect Zn QTLs was conducted to characterize candidate genes and *cis*-regulatory elements.

## Materials and methods

### Materials

All the four recombinant inbred lines (RILs) populations were developed using high Zn *aus* donor parent Kaliboro::IRGC77201-1 (P2), while promising Zn breeding lines developed at International Rice Research Institute (IRRI) were used as recipient parents viz; IR14M141 (P1), IR14M110 (P3), IR14M125 (P4), and IR95044:8-B-5-22-19-GBS (P5) (**Table S1**).

### Field establishment and phenotyping

All the mapping populations were grown in an alpha lattice design with two replications at Zeigler Experimental Station at IRRI during the dry and wet seasons of 2017 (DS2017 and WS2017) and in the dry season of 2018 (DS2018). Seeds were sown in the seedbed, and 21-day old seedlings were transplanted in the field at 20 cm x 20 cm planting distance. Each plot consisted of 2 rows with 20 hills per plot. Inorganic nitrogen (N), phosphorus (P), and potassium (K) fertilizers were applied at the rate of 120:30:30 NPK kg ha^-1^ during the DS and 90:30:30 NPK during the WS. Standard crop management practices were employed to ensure good crop growth.

We collected data on days to 50% flowering (DF), plant height (PH), number of tillers (NT), number of productive tillers (PT), panicle length (PL), thousand-grain weight (TGW), grain length (GL), grain width (GW) and grain yield per hectare (GY). We followed the standard evaluation system to gather data (IRRI, 2014). The Fe and Zn concentrations in the rice grains was measured using 3 grams of milled rice per sample by using XRF-Bruker S2 Ranger. Samples were analyzed twice, and mean values were considered for statistical analysis.

### Statistical analysis of phenotypic traits

Basic statistical analyses were performed using STAR v.2.0.1 (http://bbi.irri.org), PBTools v1.4 (http://bbi.irri.org), and R v3.5.2 in R Studio (RStudio Team, 2015). Best linear unbiased prediction (BLUP) values for each of the traits across three seasons were computed using PBTools v1.4, and were used for further analyses. Pearson’s correlation, histograms, analysis of variance (ANOVA), and descriptive statistics of traits were performed using R v3.5.2 in R Studio v1.0.153 (RStudio Team, 2015). Broad-sense heritability (H^2^) of all the traits was estimated using following formula:


$$H^{2}\;=\;\frac{\sigma_{g}^{2}}{\sigma_{p}^{2}}$$

Where σ^2^_g_ is the genotypic variance, σ^2^_p_ is the phenotypic variance,

### DNA extraction and genotyping

Fresh leaf samples were collected from RILs and parental lines during the early seedling stage. Samples were ground after freezing in liquid nitrogen and genomic DNA was extracted from each sample using the modified CTAB method (Murray and Thompson, 1980), and the quality of DNA was estimated by using 1% agarose gel electrophoresis. High-quality DNA samples with desirable concentration (~50 ng) were submitted to IRRI-Genotyping Services Laboratory (GSL) for SNP genotyping using the 7K Infinium Chip (Morales et al., 2020).

### SNP genotypic data analysis

A set of 7,086 high-quality SNP markers were generated after thorough filtering using Illumina’s protocol based on >80% call rate, homozygosity, and polymorphism between respective parents. SNP markers were further filtered based on homozygosity of the parents and with high heterozygosity on the segregating families. There were 495 high quality SNPs selected based on the distribution across the genome with < 20% missing SNPs among the parental lines. The selected SNPs were used to generate individual genetic maps and merged to construct a consensus physical map using BioMercator v4.2.1 considering a conversion of 250 kb = 1 cM. The scoring of alleles for the combined populations was performed by comparing parental alleles for each SNP. Four recipient parents were scored as “A”, donor allele was scored as “B”, and ambiguous/missing data were scored as “X”.

### Multi-cross QTL (MC-QTL) analysis

Combined phenotypic (BLUP) and genotypic data of four populations were used to perform MC-QTL analysis using MC-QTL v1.0 (Jourjon et al., 2005; De Oliviera et al., 2016). A linear regression model and iterative QTL mapping method (iQTLm) was used to detect QTL (Haley and Knott, 1992; Charcosset et al., 2000). A logarithm of odds (LOD) threshold of ≥5 was used to declare putative QTLs (Churchill and Doerge, 1994). The Phenotypic variance (R^2^) for each QTL and global R^2^ for each trait were generated. Epistatic analysis was also performed.

### QTL detection using inclusive composite interval mapping (ICIM)

QTL detection for each individual bi-parental mapping population was conducted using inclusive interval mapping (ICIM) with the aid of IciMapping v.4.1 (Wang, 2009). Critical threshold value for QTL detection was calculated by 1000 random permutations of the phenotypic data to establish an experiment-wise significance value at 0.05 (Churchill and Doerge, 1994). Estimated phenotypic variance explained by QTL for each trait and corresponding additive effect was also generated.

### Candidate gene analysis

Consistent major effect QTLs with overlapping regions and with consensus boundaries of ≤500 Kb, found across different types of QTL analysis were subjected to candidate gene analysis. Predicted candidate genes were searched within or near ( ± 500 Kb) the QTL for each trait using its flanking markers. Physical locations of the annotated genes were determined with the aid of the RAP-DB (http://rapdb.dna.affrc.go.jp/viewer/gbrowse/irgsp1) (accessed on March 20, 2019). Functions, ontology, and gene networks for each of the candidate genes were determined using the KNetminer (http://knetminer.rothamsted.ac.uk/Oryzasativa/) (accessed on March 20, 2019) and the Rice Genome Annotation Project (http://rice.plantbiology.msu.edu/cgi-bin/gbrowse/rice/) (accessed on March 20, 2019). The patterns of gene expression in various organs and stages were displayed using the eFP browser (http://bar.utoronto.ca/efprice/cgi-bin/efpWeb.cgi) (accessed on March 21, 2019), and the CoNekT (http://conekt.mpimp-golm.mpg.de/pub/) (accessed on March 21, 2019). Further searches on the previously reported QTL that co-localize with the QTL identified in our study were made using the Gramene (http://archive.gramene.org/qtl/) (accessed on March 22, 2019).

### *In silico* gene prediction and *cis*-regulatory elements analysis of grain Zn QTL

SNPs within the candidate genes related to Zn homeostasis were downloaded from the rice SNP-Seek 18 (http://snp-seek.irri.org) (accessed on March 22, 2019). Coordinates of the genomic regions of interest were identified using the reference genome (Nipponbare) by extracting flanking sequences and aligning these to the target reference genome assemblies of IR64 and Kaliboro 600 (representing the genomes of the parents). A region in the target genome with best flank hits was then extracted and characterized. Alternative SNPs between *indica*-IR64 (representing the recipient parents) and *aus*-Kaliboro 600 (representing the donor parent) were identified using Rice SNP-Seek 18 (Mansueto et al., 2017). SNP genotyping data were sourced from 3K Rice Genome Project (The 3,000 rice genome project, 2014; Wang et al., 2018). The 1 Kb upstream region of each gene was used to shortlist the SNPs in their promoter regions. All polymorphic SNPs in the promoter and coding regions were listed.

Predicted genes showing SNP polymorphisms between the query genomes were further subjected to *cis*-regulatory analysis. The 1Kb region from the coding sequences in the 5’ upstream strand of the Zn homeostasis gene was scanned for putative *cis*-regulatory elements using PlantPAN (http://PlantPAN.mbc.nctu.edu.tw) (accessed on March 23, 2019).

## Results

### Phenotypic analysis of connected populations

**Figure 1:**
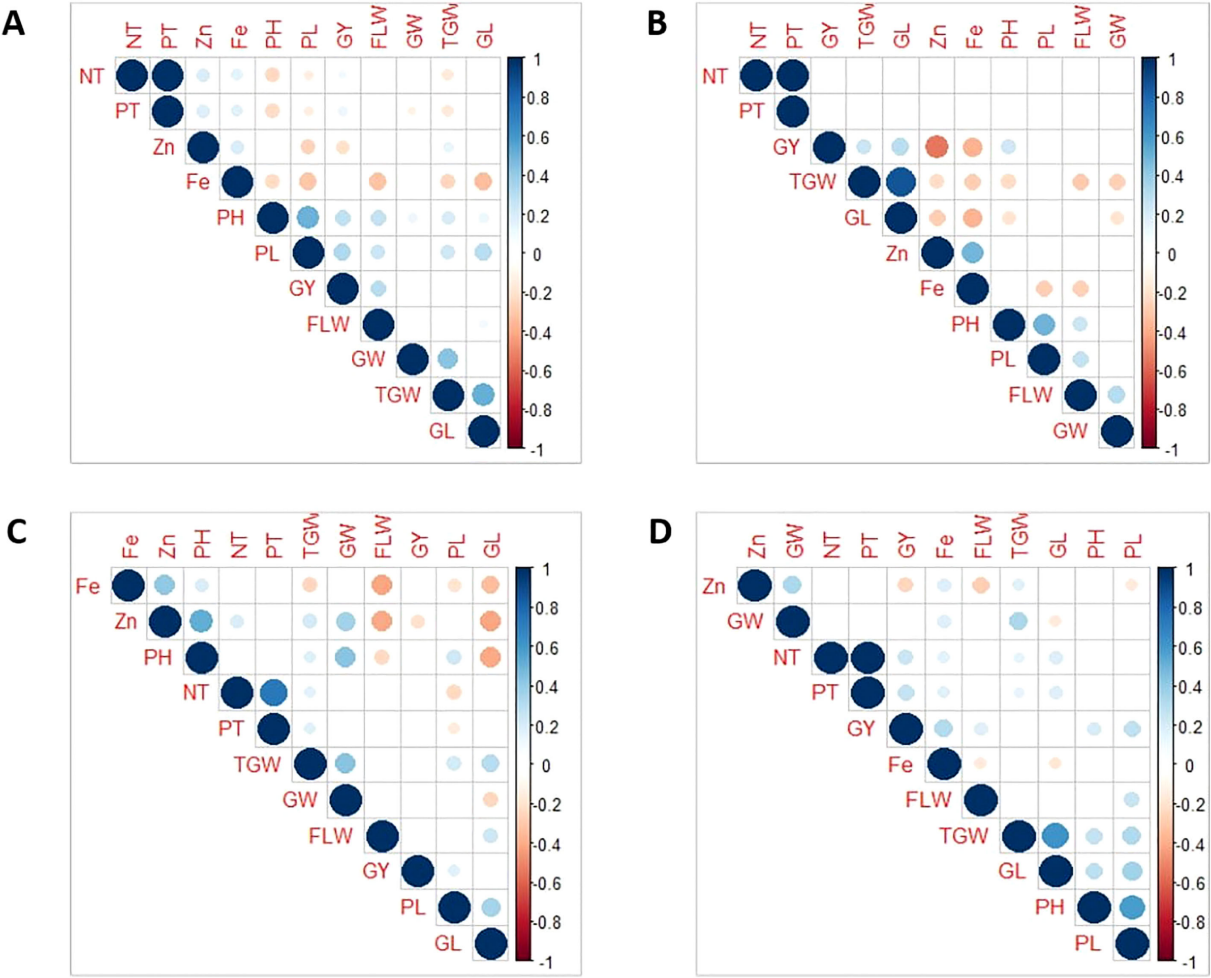
Pearson’s correlation coefficients of 11 traits in four connected populations: Pop1 **(A)**, Pop2 **(B)**, Pop3 **(C)**, and Pop4 **(D)**. GY, Grain Yield; DF/FLW, Days to flowering; PH, Plant Height; NT, Number of Tillers; PT, Productive Tillers; PL, Panicle Length; TGW, Total Grain Weight; GL, grain length; GW, Grain width.

The data of the various traits from four populations evaluated over three seasons are presented in **Table 1**. All the traits had large phenotypic variations and showed typical normal distribution across seasons and populations (**Table 1** and **Figures S1, S2**). The highest mean values were recorded for GY, TGW, and GW during the DS; while DF, PH, and PL were higher during the WS. Grain Zn and Fe concentration were high during the DS. A low coefficient of variation (CV, <10%) was observed for DF and GL, whereas a moderate to high CV (10-45%) was observed for other traits. High broad sense heritability (H^2^) was observed for Zn, DF, PH, and TGW (>70%) while it was low to moderate (10 to 60%) for all other traits (**Table 1**). Zn exhibited moderate to strong negative correlation with GY across populations (**Figures 1A–D**). Likewise, it has moderate negative correlation with TGW and GL in Pop2 (**Figure 1B**), and strong negative correlation with DF in Pop3 (**Figure 1C**). Similarly, Fe was negatively correlated with several agronomic traits: PH, PL, DF, TGW, and GL in Pop1, Pop2 and Pop3 (**Figures 1A–C**), while showed positive correlation with GW, NT, PT and GY in Pop4 (**Figure 1D**). Meanwhile, a strong positive correlation between NT, PT, TGW, and GL; with PH and PL were observed consistently across populations (**Figures 1A–D**). Notable positive correlations were observed among agronomic traits such as TGW, GW, PH, DF, and GL. The grain Fe and Zn concentration had consistent positive correlation across populations (**Figures 1A–D**).

**Table 1:** Summary statistics of phenotypic traits of four RILs across seasons.

Trait	Pop	Range			Mean ± SD			%CV			Heritability		
		17DS	17WS	18DS	17 DS	17WS	18DS	17 DS	17WS	18DS	17 DS	17WS	18DS
Zn	POP1	10.25-41.10	4.75-36.35	8.50-35.70	20.22 ± 4.45	15.97 ± 4.15	18.17 ± 4.6	22.00	26.00	25.31	0.89	0.84	0.87
	POP2	10.40-38.40	9.15-31.65	10.80-31.80	19.15 ± 4.68	16.23 ± 4.28	19.57 ± 4.34	24.43	26.37	21.17	0.94	0.84	0.93
	POP3	8.75-46.30	5.40-30.70	7.05-37.50	21.51 ± 7.92	14.80 ± 5.16	18.82 ± 6.52	36.82	34.86	27.41	0.97	0.92	0.94
	POP4	7.15-38.50	7.00-25.75	9.45-34.90	18.66 ± 4.78	13.45 ± 3.19	19.28 ± 4.52	25.61	23.71	23.44	0.79	0.79	0.83
	P1	–	–	–	13.1	10.6	14.26	–	–	–	–	–	–
	P2	–	–	–	36.49	32.59	32.79	–	–	–	–	–	–
	P3	–	–	–	12.00	10.90	11.00	–	–	–	–	–	–
	P4	–	–	–	18.98	13.55	16.83	–	–	–	–	–	–
	P5	–	–	–	16.06	11.39	16.58	–	–	–	–	–	–
Fe	POP1	1.8-21.75	0.05-9.25	1.60-10.30	5.18 ± 1.22	3.06 ± 1.21	4.87 ± 1.03	23.55	39.54	21.15	0.30	0.37	0.20
	POP2	1.4-10.50	1.05-7.10	2.00-12.5	5.13 ± 1.08	3.51 ± 0.97	3.94 ± 1.02	21.05	31.69	25.89	0.58	0.62	0.24
	POP3	1.9-17.35	0.15-5.15	1.25-9.0	5.30 ± 1.30	2.36 ± 1.08	4.18 ± 0.86	24.52	45.37	20.57	0.50	0.42	0.59
	POP4	0.80-12.10	0.20-10.10	1.50-6.70	4.77 ± 1.25	2.73 ± 1.15	4.22 ± 0.80	26.21	42.12	18.95	0.11	0.46	0.32
	P1	–	–	–	4.25	2.9	4.50	–	–	–	–	–	–
	P2	–	–	–	5.58	4.29	4.23	–	–	–	–	–	–
	P3	–	–	–	4.85	3.25	4.00	–	–	–	–	–	–
	P4	–	–	–	7.13	1.68	4.48	–	–	–	–	–	–
	P5	–	–	–	5.44	3.11	4.79	–	–	–	–	–	–
GY	POP1	1313-10803	192-9274	226-1006	4235 ± 1294	2653 ± 1157	2838 ± 1293	30.55	43.61	45.56	0.55	0.49	0.50
	POP2	133.22-9603	905-8168	834-7152	5071 ± 1618	3439 ± 1322	3313 ± 1106	31.90	38.44	33.38	0.70	0.42	0.64
	POP3	1469-10806	166-6590	1647-95705	4940 ± 1523	2928 ± 927	4956 ± 1644	30.83	26.96	33.17	0.47	0.46	0.60
	POP4	999-8809	254-6017	771-7713	3964 ± 1366	2605 ± 954	3126 ± 1073	34.46	36.61	34.32	0.59	0.62	0.56
DF	POP1	69-107	72-105	69-107	86.79 ± 7.95	87.25 ± 5.49	86.81 ± 7.96	9.16	6.29	9.17	0.99	0.76	0.65
FLW	POP2	73-101	79-101	75-95	86.17 ± 6.29	89.66 ± 5.42	84.02 ± 4.75	7.30	6.06	5.60	0.98	0.93	0.93
	POP3	76-108	73-108	69-97	89.74 ± 7.36	89.95 ± 5.93	86.6 ± 3.83	8.20	6.59	4.42	0.95	0.96	0.74
	POP4	71-103	80-109	73-93	83.69 ± 5.95	88.37 ± 5.50	82.43 ± 2.90	7.10	6.22	3.76	0.81	0.79	0.58
PH	POP1	72-181	68-187	69-164.70	122.29 ± 20.46	129.87 ± 20.94	113.68 ± 19.16	16.73	16.12	16.85	0.96	0.85	0.73
	POP2	68-181	82.83-174	75-165	119 ± 20	125.49 ± 19.52	144.55 ± 18.75	16.80	15.56	12.97	0.82	0.90	0.88
	POP3	84-172	73-168	69-166	116.42 ± 21.32	117.18 ± 23.40	109.25 ± 20.14	18.31	19.97	18.43	0.88	0.73	0.86
	POP4	64.3-185.3	61.7-186	67-151	107.64 ± 19.59	111.88 ± 19.44	102.74 ± 17.76	18.19	17.38	17.29	0.89	0.68	0.75
NT	POP1	8-25	7-23	6-26	14.57 ± 2.92	13.20 ± 1.78	11.50 ± 2.64	20.00	13.48	29.95	0.38	0.32	0.17
	POP2	5-23	8-38	7-20	13.38 ± 3.27	15.75 ± 3.81	11.42 ± 2.39	24.43	24.19	20.92	0.22	0.39	0.28
	POP3	8-24	7-45	6-48	14.16 ± 3.45	13.62 ± 3.41	12.07 ± 2.82	24.36	25.06	23.36	0.21	0.14	0.18
	POP4	6-34	5-22	4-24	13.38 ± 2.40	12.68 ± 3.02	11.30 ± 3.02	17.93	23.81	26.72	0.47	0.12	0.28
PT	POP1	8-25	7-23	6-26	14.28 ± 2.87	12.95 ± 1.76	11.58 ± 2.70	20.09	13.59	23.31	0.33	0.32	0.18
	POP2	5-23	8-38	7-20	13.16 ± 3.20	15.53 ± 3.74	11.39 ± 2.42	24.31	24.08	21.25	0.19	0.40	0.27
	POP3	5-32	7-26	6-24	14.64 ± 4.43	13.01 ± 2.92	12.36 ± 4.31	30.25	22.44	34.87	0.06	0.04	0.18
	POP4	8-24	5-22	4-24	13.26 ± 2.39	12.41 ± 3.0	11.22 ± 3.0	18.02	24.17	26.74	0.48	0.13	0.27
PL	POP1	16-32	17-35	16-32.4	23.93 ± 2.59	24.32 ± 2.91	23.92 ± 2.61	10.82	11.97	20.91	0.79	0.53	0.60
	POP2	9.8-32.2	17.33-33	18-28	25.22 ± 2.56	24.65 ± 2.52	22.5 ± 1.79	10.15	10.22	7.96	0.79	0.61	0.32
	POP3	17-30	19-30	16-36	24.25 ± 2.20	25.21 ± 1.97	24.82 ± 2.23	9.07	7.80	8.98	0.55	0.43	0.42
	POP4	15.84-29.86	16.3-30.7	16.33-29.67	22.29 ± 2.40	23.70 ± 3.0	24.32 ± 2.76	10.76	12.65	11.35	0.82	0.40	0.50
TGW	POP1	19.00-40.00	15.60-32.30	20.90-37.30	26.47 ± 3.00	24.13 ± 2.74	27.83 ± 2.80	11.33	11.36	10.06	0.83	0.81	0.70
	POP2	20.04-31.72	18.00-39.20	15.60-32.80	25.77 ± 2.12	24.27 ± 2.57	24.75 ± 3.02	8.23	10.59	12.20	0.75	0.65	0.69
	POP3	13.60-31.12	15.9-35.9	5.28-34.68	25.87 ± 2.95	26.17 ± 2.13	26.51 ± 2.83	11.40	8.13	10.67	0.86	0.80	0.76
	POP4	8.08-34.20	20.80-32.8	16.32-39.68	24.43 ± 3.13	26.54 ± 2.66	25.11 ± 3.58	12.81	10.02	14.25	0.89	0.19	0.55
GL	POP1	7.47-11.10	7.82-11.73	7.60-11.1	9.25 ± 0.65	9.29 ± 0.59	9.17 ± 0.63	7.07	6.30	6.87	0.89	0.84	0.80
	POP2	7.74-10.94	7.89-11.70	7.77-10.78	9.39 ± 0.65	9.39 ± 0.68	9.58 ± 0.64	5.90	7.24	6.68	0.92	0.84	0.89
	POP3	7.48-18.74	8.03-8.79	8.13-10.79	8.88 ± 0.78	9.35 ± 0.55	9.46 ± 0.60	8.78	5.88	6.34	0.75	0.91	0.87
	POP4	6.73-10.99	7.60-10.65	7.43-10.98	8.76 ± 0.70	9.16 ± 0.59	9.19 ± 0.70	7.99	6.44	7.16	0.89	0.86	0.83
GW	POP1	2.28-3.54	1.75-2.21	2.20-8.70	2.90 ± 0.24	1.98 ± 0.08	2.77 ± 0.31	8.27	4.04	11.19	0.71	0.72	0.49
	POP2	2.24-3.22	1.74-3.53	2.18-4.73	2.72 ± 0.22	1.99 ± 0.22	2.65 ± 0.31	8.09	11.06	11.70	0.78	0.69	0.41
	POP3	1.90-4.32	2.17-3.66	2.23-6.92	2.47 ± 0.38	2.83 ± 0.24	2.77 ± 1.25	15.38	8.40	15.15	0.73	0.10	0.16
	POP4	1.92-3.03	2.15-3.10	1.35-3.34	2.41 ± 0.26	2.57 ± 0.17	2.50 ± 0.36	10.79	6.66	14.40	0.65	0.69	0.33

GY, Grain Yield; DF/FLW, Days to flowering; PH, Plant Height; NT, Number of Tillers; PT, Productive Tillers; PL, Panicle Length; TGW, Total Grain Weight; GL, grain length; GW, Grain width; Pop, Population; Pop1, Population 1; Pop2, Population 2; Pop3, Population 3; Pop4, Population 4; DS, Dry Season; WS, Wet Season; CV, Coefficient of Variation; SD, Standard Deviation; -, no data available and not applicable.

### Multi-cross QTL analyses of connected populations

Overall, 495 SNPs across four populations were used to construct a consensus genetic map and used for MC-QTL analyses. The number of SNPs per chromosome ranged from 11 to 75 with lowest and highest number of SNPs on chromosomes 9 and 1, respectively (**Figure S5**). The total map length was 1627 cM with an average distance of 6.0 cM between SNPs. MC-QTL analyses identified MC-156 QTLs for 11 traits (**Table 2**). The global R^2^ captured by the QTLs varied from 12 to 58% for various traits, while the R^2^ explained by individual QTL ranged from 0.1 to 22%. The two major QTLs with highest R^2^ (22% and 13%, respectively) were *qPH_1.1_* and *qZn_5.2_*. The tall parent (P2) allele increased PH by 7.19 cm while the other parents reduced PH by 1.77 to 1.91 cm. Some of the major QTLs for agronomic and yield traits were: *qDF_7.1_*, *qNT_4.1_*, *qPT_4.1_*, *qPL_1.2_*, *qTGW_5.1_*, *qGL_3.1,_ qGW_6.1_* and *qGY_10.1_* (**Table 2**). The *qDF_7.1_* located between id7005418 and c7p27670416 on chromosome 7 explained 7.3% R^2^, P1 allele increased maturity by 1.70 days while P5 allele reduced maturity by 0.80 days. Whereas, *qNT_4.1_* and *qPT_4.1_* were detected between 3647133 and 3653113 on chromosome 4, each explained by a R2 >3%. Meanwhile, *qPL_1.2_* detected on chromosome 1 explained 7% R^2^, and *qTGW_5.1_* identified between 5020568 and 5056493 increased TGW by 0.52 g. Among the 21 QTLs identified for GL, *qGL_3.1_* explained 8% R^2^, P1 allele increased GL by 0.09 mm. For grain width, *qGW_6.1_* contributed 4.2% R^2^ and P3 allele increased grain width 0.03 mm. The *qGY_10.1_* explained 4% R^2^ with highest positive allelic contribution from P2 (85.5 kg/ha).

**Table 2:** QTL detected for Zn, Fe, GY, and GY-related traits of four connected populations using MC-QTL.

Trait	No. of QTL	QTL Name	Chr		QTL Position (Mb)	Right Marker		Left Marker	% R^2^ Value	% R^2^ Global	Additive Effects					
											P1	P2	P3	P4	P5	
Zn	1	*qZn_1.1a_*		1	2.46	105375		107614	1.7	58	-0.14	0.17	0.62	-0.96	0.32
	2	*qZn_1.1b_*		1	22.10	SNP-1_22828195		id1013342	1.3		0.32	0.34	-0.62	-0.20	0.16
	3	*qZn_1.2_*		1	37.50	1225497		1226391	0.4		0.15	0.10	-0.24	0.18	-0.28
	4	*qZn_2.1_*		2	11.92	id2005552		1751938	0.3		0.19	0.07	0.05	-0.36	0.05
	5	*qZn_2.2_*		2	34.41	SNP-2.34686373.			1.2		-0.27	-0.30	-0.75	-0.17	0.00
	6	*qZn_3.1_*		3	6.45	id3004358		2703814	1.6		-0.34	0.14	0.48	0.20	-0.47
	7	*qZn_3.2_*		3	24.13	3285365		id3011085	4.3		0.09	0.36	1.12	-1.70	0.14
	8	*qZn_4.1_*		4	0.59	3606371		SNP-4.955413.	0.8		-0.17	0.21	0.11	-0.08	-0.07
	9	*qZn_4.2_*		4	12.39	4119916		SNP-4.12588754.	0.4		-0.05	-0.01	-0.37	0.45	-0.02
	10	*qZn_4.2_*		4	32.45	4743351		4761773	0.4		-0.02	0.16	0.09	-0.03	-0.20
	11	*qZn_5.1_*		5	5.17	5140013		SNP-5.9394949.	3.3		0.31	0.60	0.22	-1.02	-0.11
	12	*qZn_5.2_*		5	22.03	5711540		5726844	13.0		-1.25	0.47	1.06	-0.73	0.44
	13	*qZn_6.1_*		6	0.00	5965570		6033802	2.8		0.13	0.32	-1.00	-0.29	0.84
	14	*qZn_6.2_*		6	18.87	id6012531		SNP-6.24472951.	2.1		-0.32	0.19	-0.99	0.74	0.37
	15	*qZn_7.1_*		7	7.61	id7001824		id7001964	0.3		0.00	-0.11	0.03	0.39	-0.31
	16	*qZn_7.2_*		7	24.99	c7p27670416		7932889	2.4		0.00	0.48	-0.57	-0.28	0.36
	17	*qZn_8.1_*		8	0.00	id8000315		8024868	0.4		-0.23	-0.20	0.25	0.20	-0.02
	18	*qZn_8.2_*		8	16.96	SNP-8.17813578.		8815450	0.8		0.12	-0.14	0.45	-0.62	0.19
	19	*qZn_8.2_*		8	27.52	id8007977			0.3		0.02	0.02	-0.37	0.32	0.01
	20	*qZn_9.1_*		9	7.12	id9000710		9569595	2.5		-0.09	-0.65	0.23	0.40	0.11
	21	*qZn_9.2_*		9	20.49	9851330			0.5		0.25	0.20	0.01	-0.62	0.16
	22	*qZn_10.1_*		10	1.44	SNP-10.2441725.		id10000771	1.1		0.33	-0.15	-0.22	-0.13	0.16
	23	*qZn_10.2_*		10	20.14	id10006640		10778744	0.7		0.08	-0.04	-0.25	-0.64	0.58
	24	*qZn_11.1_*		11	0.43	SNP-11.4183712.		10944235	3.1		0.16	0.26	1.26	-1.52	0.16
	25	*qZn_11.2_*		11	14.47	11514349		11619512	1.6		-0.10	-0.19	-0.69	1.20	-0.22
	26	*qZn_12.1_*		12	1.62	12048140		id12001059	0.4		-0.28	0.02	0.13	-0.08	0.20
	27	*qZn_12.2_*		12	21.70	SNP-12.21800911.		id12008917	3.0		-0.06	0.33	0.62	0.95	-0.03
Fe	1	*qFe_1.1_*		1	2.46	105375		107614	0.7	25	-0.01	0.04	0.00	-0.06	0.02
	2	*qFe_2_._1_*		2	23.99	SNP-2.24266178.		id2011968	3.0		0.09	0.00	0.16	-0.06	-0.19
	3	*qFe_3_._1_*		3	14.53	2915115		2916182	2.1		-0.04	0.07	0.02	0.04	-0.07
	4	*qFe_4.1_*		4	6.39	3911347		id4002942	1.4		0.00	0.00	-0.15	0.04	0.11
	5	*qFe_4_._2_*		4	25.96	4560749		4610790	0.7		-0.05	-0.03	0.06	0.09	-0.07
	6	*qFe_6.1_*		6	2.02	6033802		6048208	0.8		-0.03	-0.02	-0.07	0.08	0.03
	7	*qFe_7.1_*		7	1.68	id7000589		7072228	1.0		0.08	0.01	-0.08	0.02	-0.03
	8	*qFe_7.2_*		7	21.00	7839126		id7005418	3.2		-0.07	0.07	-0.10	0.04	0.07
	9	*qFe_8.1_*		8	0.00	id8000315		8024868	2.3		-0.09	0.01	-0.10	0.09	0.09
	10	*qFe_8.2_*		8	16.96	SNP-8.17813578.		8815450	3.0		0.00	-0.01	0.05	-0.05	0.03
	11	*qFe_9.1_*		9	7.12	id9000710		9569595	1.8		0.02	-0.06	0.07	-0.14	0.11
	12	*qFe_11.1_*		11	5.65	SNP-11.9404381.		11223687	1.0		0.07	0.01	-0.03	0.03	-0.09
	13	*qFe_12.1_*		12	4.75	c12p4887439		12159979	2.4		0.06	0.03	0.02	0.06	-0.17
	14	*qFe_12.2_*		12	25.66	SNP-12_25758809			3.0		-0.04	0.00	0.03	-0.02	0.04
GY	1	*qGY_1.1_*		1	6.90	id1005511	237468		2.3	32	73.00	-80.0	141.0	-45.00	-88.00
	2	*qGY_1.2_*		1	22.61	id1013342	SNP-1.23987271.		0.1		-42.07	3.50	-118.4	90.30	66.60
	3	*qGY_1.2_*		1	39.07	id1025292	1286531		2.4		-97.90	65.1	-83.70	43.10	73.40
	4	*qGY_2.1_*		2	2.95	id2001831	SNP-2.3340604.		2.4		-3.20	-30.5	-179.5	16.90	196.3
	5	*qGY_3.1_*		3	7.61	2703814	SNP-3.9896907.		1.3		-63.90	40.0	-93.40	18.50	101.80
	6	*qGY_4.2_*		4	32.45	4743351	4761773		0.2		35.90	25.5	-39.80	-0.76	-20.90
	7	*qGY_5.1_*		5	2.47	5020568	5056493		0.5		10.20	6.26	-120.5	60.30	43.60
	8	*qGY_6.1_*		6	2.34	6048208	SNP-6.8062552.		3.0		-9.30	-74.6	281.7	-121.6	-76.20
	9	*qGY_6.2_*		6	24.17	6907224	id6016547		1.2		-99.40	-24.9	94.10	7.95	82.20
	10	*qGY_7.1_*		7	4.52	ud7000557	7230009		1.3		51.10	-27.9	6.50	91.40	-121.8
	11	*qGY_7.2_*		7	25.56	7946044	id7005665		0.8		81.60	12.9	10.50	-7.60	-97.40
	12	*qGY_8.1_*		8	25.94	SNP-8.26796600.	9035961		2.0		106.2	-29.4	-35.50	-31.90	-9.40
	13	*qGY_9.1_*		9	7.12	id9000710	9569595		2.4		-142.9	27.7	93.40	-82.20	158.0
	14	*qGY_10.1_*		10	8.06	SNP-10.8991939.	id10002542		4.0		-62.10	85.5	75.90	-14.80	-84.30
	15	*qGY_11.1_*		11	0.38	SNP-11.4136091.	SNP-11.4183712.		1.5		39.40	-19.0	-205.7	100.8	84.50
	16	*qGY_11.2_*		11	25.22	SNP-11.28451186.			0.8		46.60	21.7	-159.2	61.50	29.40
	17	*qGY_12.1_*		12	16.26	SNP-12.16391600.	SNP-12.17624959.		1.4		13.40	-41.6	129.0	41.40	-142.2
DF/FLW	1	*qDF_1.1_*		1	29.59	1006459	id1018601		0.8	20	-0.07	-0.34	-0.29	0.74	-0.10
	2	*qDF_3.1_*		3	0.00	2519506	id3001137		4.0		-0.94	0.56	-0.60	0.88	0.09
	3	*qDF_5.2_*		5	24.99	5786385			1.4		0.45	-0.42	-0.53	0.60	-0.10
	4	*qDF_7.1_*		7	24.90	id7005418	c7p27670416		7.3		1.70	-0.68	0.33	-0.55	-0.80
	5	*qDF_9.1_*		9	0.00	SNP-9.795433.	id9000710		3.0		-0.93	0.35	-0.96	0.84	0.69
	6	*qDF_10.1_*		10	14.36	10585903	10651050		3.0		-1.16	0.36	0.72	0.57	-0.27
	7	*qDF_12.1_*		12	21.70	SNP-12.21800911.	id12008917		0.6		0.00	0.34	0.81	0.28	0.20
PH	1	*qPH_1.1_*		1	38.35	1250974	SNP-1.39133884.		22.0	43	-1.76	7.19	-1.77	-1.91	-1.74
	2	*qPH_2.1_*		2	10.95	1717430	id2005552		1.1		-0.10	-0.88	4.03	-2.15	-0.89
	3	*qPH_2.2_*		2	34.41	SNP-2.34686373.			1.4		-0.82	1.30	-1.62	1.54	-0.39
	4	*qPH_3.1_*		3	0.00	2519506	id3001137		0.9		1.53	0.52	-1.10	1.42	-2.34
	5	*qPH_4.1_*		4	0.00	SNP-4.203305.	3606371		2.0		1.69	-0.73	-1.12	-1.88	2.04
	6	*qPH_4.2_*		4	29.36	4653224	4657360		1.7		0.34	1.16	-2.12	-4.00	4.62
	7	*qPH_5.1_*		5	22.64	5726844	SNP-5.27621108.		0.9		0.66	1.41	-2.79	0.34	0.37
	8	*qPH_6.1_*		6	6.21	id6007220	6228191		0.9		-0.66	0.99	1.25	-1.70	0.12
	9	*qPH_7.1_*		7	9.09	7370977	7452767		0.7		1.36	0.12	-2.76	0.99	0.39
	10	*qPH_8.1_*		8	16.96	SNP-8.17813578.	8815450		1.4		-0.10	-1.06	1.32	-2.97	2.81
	11	*qPH_9.1_*		9	13.13	9627787	c9p13985040		1.9		-2.96	0.28	1.74	0.11	0.83
	12	*qPH_10.1_*		10	14.36	10585903	10651050		1.9		-0.40	1.08	2.81	-3.59	0.10
	13	*qPH_12.1_*		12	2.15	id12001059	id12001156		2.0		0.11	0.66	-0.83	0.20	-0.14
NT	1	*qNT_3.1_*		3	2.82	2584928	id3002805		1.4	13	-0.18	-0.01	0.00	0.07	0.13
	2	*qNT_4.1_*		4	1.51	3647133	3653113		3.1		0.01	0.08	0.09	0.09	-0.27
	3	*qNT_4.2_*		4	28.59	4629297	4639043		2.0		0.04	0.05	-0.32	-0.10	0.32
	4	*qNT_8.1_*		8	0.00	id8000315	8024868		1.4		-0.10	0.04	-0.13	0.01	0.18
	5	*qNT_9.1_*		9	15.03	SNP-9.15828930.	9753048		1.2		-0.08	0.00	-0.11	-0.03	0.22
	6	*qNT_11.1_*		11	5.55	SNP-11.9300050.	SNP-11.9404381.		1.6		0.18	0.05	0.05	-0.11	-0.17
PT	1	*qPT_3.1_*		3	2.81	2584928	id3002805		1.3	12	-0.16	-0.02	-0.01	0.05	0.14
	2	*qPT_4.1_*		4	1.51	3647133	3653113		4.0		0.01	0.08	0.09	0.11	-0.29
	3	*qPT_4.2_*		4	28.59	4629297	4639043		2.1		0.03	0.04	-0.35	-0.01	0.30
	4	*qPT_8.1_*		8	0.00	id8000315	8024868		1.4		-0.10	0.05	-0.11	0.00	0.16
	5	*qPT_9.1_*		9	15.03	SNP-9.15828930.	9753048		1.9		-0.10	0.00	-0.10	-0.07	0.26
PL	1	*qPL_1.1_*		1	1.56	74775	105375		1.4	31	0.17	-0.04	0.02	0.06	-0.22
	2	*qPL_1.2_*		1	37.11	SNP-1.37834974.	1225497		7.0		-0.21	0.29	-0.02	0.08	-0.13
	3	*qPL_2.1_*		2	2.95	id2001831	SNP-2.3340604.		1.7		0.01	-0.11	-0.21	0.17	0.15
	4	*qPL_3.1_*		3	0.00	2519506	id3001137		1.5		0.25	0.03	-0.05	-0.03	-0.20
	5	*qPL_3.2_*		3	13.77	id3007788	SNP-3.15794383.		1.4		0.03	-0.14	-0.04	0.10	0.05
	6	*qPL_4.1_*		4	27.91	4610790	4629297		2.3		-0.21	-0.22	-0.07	0.19	0.30
	7	*qPL_5.1_*		5	0.00	4928297	4931423		2.1		0.11	-0.13	0.21	-0.22	0.04
	8	*qPL_6.1_*		6	0.00	5965570	6033802		0.8		0.10	-0.01	0.08	0.17	-0.17
	9	*qPL_9.1_*		9	0.00	SNP-9.795433.	id9000710		1.4		-0.18	0.03	-0.16	0.13	0.18
	10	*qPL_9.2_*		9	20.49	9851330			3.6		-0.07	0.14	0.19	0.11	-0.38
	11	*qPL_10.1_*		10	9.65	id10002842	10515733		0.7		-0.02	0.10	-0.08	0.03	-0.03
	12	*qPL_10.2_*		10	20.14	id10006640	10778744		1.6		-0.13	0.11	0.20	-0.15	-0.04
	13	*qPL_11.1_*		11	0.38	SNP-11.4136091.	SNP-11.4183712.		1.1		0.03	-0.06	0.28	-0.31	0.06
	14	*qPL_12.1_*		12	5.25	12172432	12271792		2.8		0.16	-0.14	-0.10	-0.11	0.19
TGW	1	*qTGW_1.1_*		1	21.79	753735	SNP-1_22828195		1.1	27	0.20	0.06	0.04	0.04	-0.34
	2	*qTGW_3.1_*		3	29.59	3437076	3456359		2.0		-0.33	0.06	0.21	0.08	-0.01
	3	*qTGW_4.1_*		4	27.91	4610790	4629297		2.8		-0.37	-0.08	-0.08	0.04	0.49
	4	*qTGW_5.1_*		5	2.47	5020568	5056493		5.0		0.52	-0.05	-0.19	-0.03	-0.25
	5	*qTGW_5.2_*		5	23.72	5754154	5786385		2.0		-0.09	0.02	0.06	-0.04	0.05
	6	*qTGW_6.1_*		6	2.02	6033802	6048208		0.8		-0.12	0.08	0.12	-0.13	0.05
	7	*qTGW_6.2_*		6	19.46	6758176	id6013529		2.6		-0.29	0.13	0.28	-0.12	0.00
	8	*qTGW_7.1_*		7	1.55	7066952	id7000589		0.5		0.00	-0.04	0.00	-0.16	0.20
	9	*qTGW_8.1_*		8	2.36	8076764	id8001139		2.1		0.31	-0.03	-0.10	-0.28	0.09
	10	*qTGW_9.1_*		9	13.13	9627787	c9p13985040		0.6		-0.03	0.10	0.13	-0.09	-0.09
	11	*qTGW_10.1_*		10	2.70	10077150	SNP-10.4699242.		0.1		0.08	0.15	0.11	-0.18	-0.16
	12	*qTGW_11.1_*		11	5.55	SNP-11.9300050.	SNP-11.9404381.		0.7		0.10	0.16	0.15	-0.30	-0.11
	13	*qTGW_12.1_*		12	2.60	id12001156	id12001775		0.1		0.00	0.03	0.06	-0.02	-686.0
GL	1	*qGL_1.1_*		1	9.61	311989	328663		0.1	41	0.06	0.00	0.01	-0.08	0.01
	2	*qGL_1.2_*		1	23.69	SNP-1.24413541.	SNP-1.24461978.		1.8		0.08	0.03	0.00	0.00	-0.11
	3	*qGL_1.2_*		1	37.92	1237300	SNP-1.38961387.		4.3		-0.07	0.04	0.02	0.14	-0.12
	4	*qGL_2.1_*		2	0.00	1374878	1412828		0.2		0.05	-0.04	-0.05	0.02	0.02
	5	*qGL_2.2_*		2	34.41	SNP-2.34686373.			0.4		0.00	-0.03	0.00	0.03	0.00
	6	*qGL_3.1_*		3	14.97	GS3	id3008386		8.0		0.09	-0.10	-0.04	0.07	-0.02
	7	*qGL_3.2_*		3	26.93	3373021	3437076		2.2		-0.08	0.03	0.03	0.00	0.03
	8	*qGL_4.1_*		4	32.45	4743351	4761773		1.2		0.01	-0.04	-0.04	0.05	0.02
	9	*qGL_5.1_*		5	2.47	5020568	5056493		1.1		0.07	0.01	0.00	-0.04	-0.05
	10	*qGL_5.2_*		5	14.81	id5007657	5536338		0.5		-0.03	-0.01	-0.02	0.09	-0.03
	11	*qGL_6.1_*		6	17.80	6702537	6728026		2.4		-0.03	0.04	0.07	0.01	-0.10
	12	*qGL_7.1_*		7	7.39	id7001787	7299668		0.7		-0.01	0.04	-0.01	0.26	0.04
	13	*qGL_7.2_*		7	24.87	id7005418	c7p27670416		1.3		0.05	-0.02	-0.05	-0.03	0.05
	14	*qGL_8.1_*		8	7.65	id8002628	8342673		0.5		0.01	0.01	0.00	-0.09	0.07
	15	*qGL_8.2_*		8	19.19	8815450	8815799		0.7		-0.03	0.00	-0.03	-0.02	0.09
	16	*qGL_9.1_*		9	7.12	id9000710	9569595		1.1		0.00	0.01	0.01	0.11	-0.12
	17	*qGL_9.2_*		9	17.51	9766886	9851330		2.1		0.05	0.04	0.05	-0.16	0.03
	18	*qGL_10.1_*		10	9.65	id10002842	10515733		0.1		-0.01	-0.04	-0.03	0.08	0.00
	19	*qGL_11.1_*		11	13.51	SNP-11.16797379.	SNP-11.17276263.		0.4		0.03	0.02	0.01	-0.02	-0.03
	20	*qGL_12.1_*		12	4.88	12159979	12172432		1.7		-0.10	-0.02	0.00	0.05	0.07
	21	*qGL_12.2_*		12	25.66	SNP-12_25758809			0.1		0.02	0.01	-0.03	-0.01	0.00
GW	1	*qGW_1.1_*		1	1.56	74775	105375		4.0	41	-0.02	-0.03	-0.04	0.08	0.00
	2	*qGW_1.2_*		1	14.21	491345	491417		1.6		0.01	0.01	0.01	-0.05	0.02
	3	*qGW_1.2_*		1	37.00	id1023824	SNP-1.37834974.		1.1		-0.01	0.00	0.00	-0.03	0.04
	4	*qGW_2.1_*		2	21.33	id2008655	2078876		2.5		0.01	0.02	0.02	-0.06	0.02
	5	*qGW_2.2_*		2	32.50	2403924	id2014833		0.4		-0.01	0.01	0.01	-0.01	-0.01
	6	*qGW_3.1_*		3	23.38	id3010971	3285365		1.3		0.00	0.02	0.00	-0.01	0.00
	7	*qGW_4.1_*		4	3.43	id4001482	ud4000438		1.0		0.00	0.01	0.02	-0.03	0.00
	8	*qGW_4.2_*		4	27.91	4610790	4629297		1.8		-0.04	-0.02	-0.01	0.07	0.00
	9	*qGW_5.1_*		5	2.47	5020568	5056493		3.1		0.01	-0.01	-0.01	0.05	-0.03
	10	*qGW_5.2_*		5	24.99	5786385			1.1		0.00	0.01	0.00	-0.04	0.03
	11	*qGW_6.1_*		6	14.97	6585321	6619487		4.2		0.02	0.02	0.03	-0.09	0.02
	12	*qGW*_7.1_		7	3.05	7110346	7140747		1.8		0.01	0.02	0.02	-0.07	0.03
	13	*qGW*_7.2_		7	21.00	7818489	7839126		0.5		0.00	-0.01	0.00	0.04	0.02
	14	*qGW*_8.1_		8	25.94	SNP-8.26796600.	9035961		0.3		-0.01	0.00	0.00	0.02	-0.01
	15	*qGW*_9.1_		9	20.49	9851330			0.7		0.00	0.00	0.00	0.00	0.00
	16	*qGW*_10.1_		10	7.69	SNP-10.8621011.	SNP-10.8991939.		2.8		0.02	0.03	0.02	-0.06	-0.01
	17	*qGW*_11.1_		11	1.26	10963017	id11003539		0.2		0.01	0.01	0.00	0.00	-0.01
	18	*qGW*_11.2_		11	14.47	11514349	11619512		0.8		-0.01	-0.01	0.00	0.05	-0.03
	19	*qGW*_12.1_		12	2.60	id12001156	id12001775		0.1		0.01	0.00	-0.01	-0.01	0.00

GY, Grain Yield; DF/FLW, Days to flowering; PH, Plant Height; NT, Number of Tillers; PT, Productive Tillers; PL, Panicle Length; TGW, Total Grain Weight; GL, grain length; GW, Grain width; Chr, Chromosome; QTL, Quantitative Trait Loci; R2, Phenotypic variance, P1, Parent 1; P2, Parent 2; P3, Parent 3; P4, Parent 4.

A total of 27 QTL was detected for Zn that explained R^2^ of 0.3 to 13.0% with a global R^2^ of 58%. As expected, the donor parent (P2) had the highest combined additive effect (2.6 ppm). Interestingly, the recipient parent (P5) total allele contribution was 2.5 ppm (**Table 2**). A major QTL (*qZn_5.2_*) with an R^2^ of 13% was detected on chromosome 5 at 22.03 Mb flanked by markers 5711540 and 5726844. It has a narrow genomic region of 600 Kb among the Zn QTLs. There were three QTLs, *qZn_3.2_, qZn_11.1a_* and *qZn_12.1b_* each contributed >3% R^2^. Similarly for Fe, 14 QTLs were detected with R^2^ ranging 0.7-3.2% and a global R^2^ of 25%. These QTL were located on all the chromosomes except chromosomes 5 and 10. The *qFe_7.2_*, located on chromosome 7 at 21 Mb, accounted for the highest R^2^ (3.2%). However, the highest positive allele contribution with 0.16 ppm increase in Fe concentration observed for *qFe_2.1_*.

### Co-location of QTL identified

**Figure 2:**
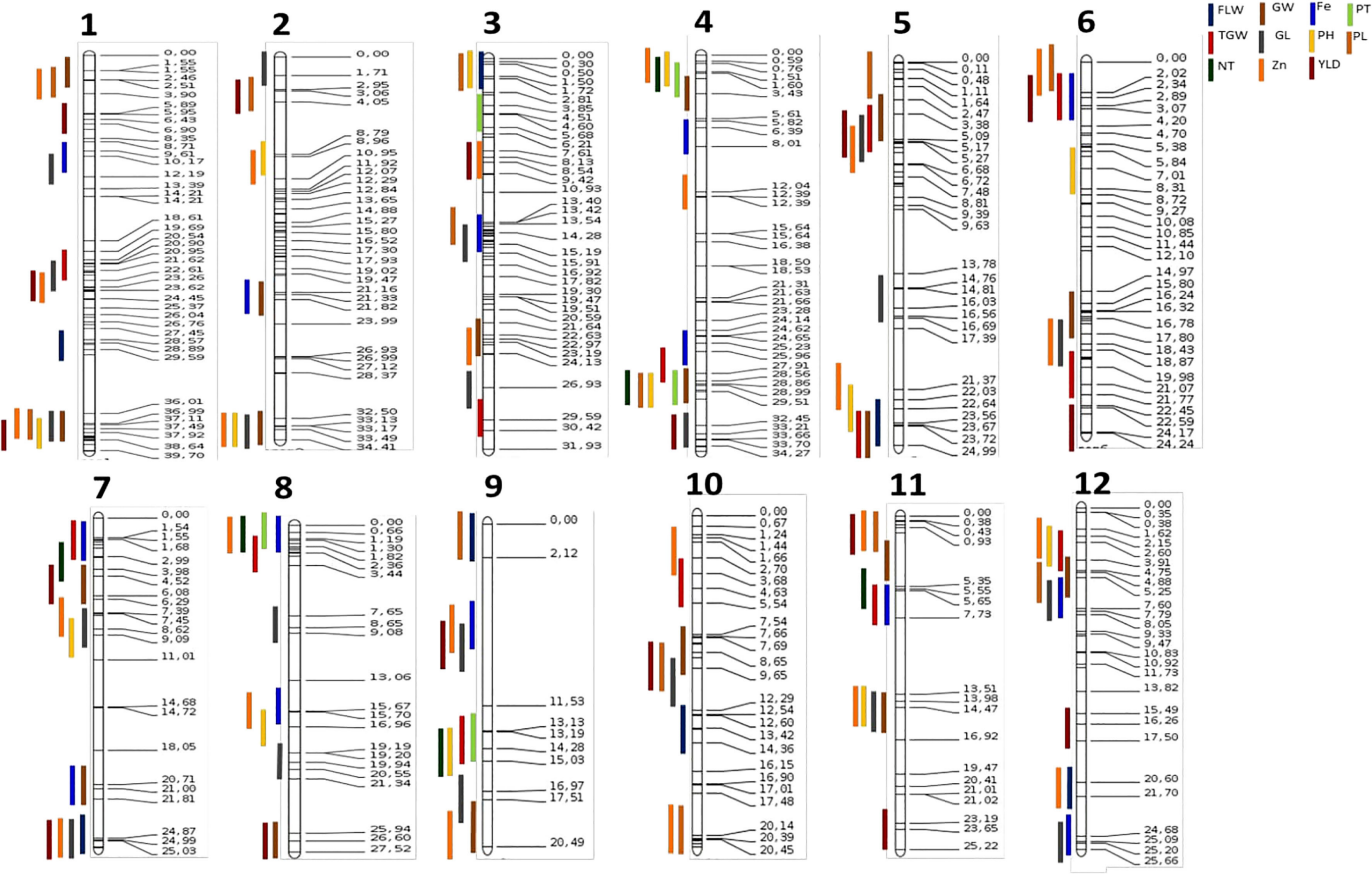
Consensus map of QTL detected for various traits. Vertical colored lines on the left represent different QTL while values on the horizontal lines at the right side show the position of the markers on the chromosomes; numbers below the figure indicate the corresponding chromosome.

QTLs that shared common SNPs were considered co-located. In all, 26 QTL clusters were identified on different chromosomes (**Figure 2**). Four QTLs, *qGY_5.1_*, *qTGW_5.1_*, *qGL_5.1_*, and *qGW_5.1_* were co-located at 2.5 Mb on chromosome 5, while QTLs *qZn_8.1_*, *qFe_8.1_*, *qNT_8.1_*, and *qPT_8.1_* were at the same genomic region on the short arm of chromosome 8. Similarly, *qGY_9.1_*, *qZn_9.1_*, *qFe_9.1_*, and *qGL_9._*_1_ co-localized between markers id9000710 and 9569595 on chromosome 9. We observed three QTLs clusters at four genomic regions on chromosomes 3, 4, 8, and 9, while 20 genomic regions had two QTLs co-located (**Figure 2**).

### Epistasis

Epistatic interactions were detected between *qZn_5.1_* and *qPH_6.1_*, and *qPH_6.1_* and *qGY_6.2_*; each explained a R^2^ of 5% (**Table S2**). *qPH_6.1_* is a minor QTL flanked by id6007220 and 6228191 with genetic interval of 8.08 Mb. The positive allele (P3) increased PH by 1.25 cm while P4 allele reduced PH by 1.70 cm. On the other hand, *qZn_5.1_* is also a minor allele associated with Zn flanked by 514403 and SNP-5.9394949 with positive allele contributed by P2 (0.60 ppm). Minor QTL, *qPH_6.1_* is also interacted with *qGY_6.2_* which was flanked by markers 6907224 and id6016547 with a narrow interval of 0.07 Mb. The positive alleles were contributed by P3 and P4 with an additive effect of 100 kg/ha (**Table 2**).

### Candidate genes for agronomic, yield and biofortification traits

Largest-effect QTL for grain yield, agronomic traits, Fe, and all the QTLs detected for grain Zn were subjected to candidate gene analysis (**Table 2** and **Figure S3**). Genes conferring biological functions related to the trait were shortlisted (**Table 3**). The *OsMADS18* is co-located with *qFe_7.1_*, it helps in Fe transport, cellular and inter-cellular responses under Fe deficiency, it is also known to regulate seed maturation and days to maturity. Similarly, *HUA2* was linked to flowering locus *qDF_7.1_* and regulates flowering time and reproductive development. A pyruvate dehydrogenase kinase gene (*OsPdk*1) is found nearest to *qPL_1.2_* which is involved in root hair length and panicle threshability. Meanwhile, Glycogen Synthase Kinase (*GSK)* is found to influence days to maturity, seed maturation, brown rice shape, grain width and grain weight. GSK2 was co-located with major effect QTL, *qTGW_5.1_*, while GSK4 was co-located with *qGW_6.1_*. Similarly, *GS3* was within the confidence interval of *qGL_3.1_*. Candidate genes *OS01G0899425, OS04G0350700*, and *OS10G0326900* were co-located with *qPH_1.1_*, *qPT_4.1_*, and *qGY_10.1_*, respectively (**Table 3**).

**Table 3:** Identified putative genes associated with major QTL for Zn, Fe, GY and grain-related traits of connected populations.

QTL	Chr	Interval (Mb)	Gene	Gene ID	Position (Mb)	Gene Ontology	Reference
*qFe_7.1_*	7	24.48-27.54	*MADS18*	*OS07G0605200*	24.78	Iron concentration, days to maturity, iron concentration, seed maturation, intracellular iron storage, cellular response to iron, iron transporter	(Wei et al., 2012; Yin et al., 2019)
*qDF_7.1_*	7	27.54-27.67	*HUA2*	*OS07G0655500*	27.55	Regulator of flowering time and reproductive development	(Chen & Meyerowitz, 1999)
*qPH_1.1_*	1	39.07-39.13		*OS01G0899425*	39.12	Plant height, disease control	(Sevanthi et al., 2021)
*qPT_4.1_*	4	17.14-18.06		*OS04G0350700*	17.23	Productive tillers	(Li et al., 2016)
*qPL_1.2_*	1	37.83-38.22	*OsPdk1*	*OS1G0872800*	37.85	Root hair length, panicle threshability	(Jan et al., 2006)
*qTGW_5.1_*	5	6.59-7.50	*GSK2*	*OS05G0207500*	6.67	Average grain weight, protein concentration, total amylase activity trait, days to maturity, potassium uptake, seed maturation, seed weight, grain width	(Hu et al., 2015)
*qGY_10.1_*	10	9.06-9.10		*OS10G0325400*	9.00	grain number, days to maturity, seed maturation, brown shape rice	
*qGL_3.1_*	3	16.73-16.95	*GS3*	*OS03G0407400*	16.73	Grain length	(Fan et al., 2006)
*qGW_6.1_*	6	20.14-20.98	*GSK4*	*OS06G0547900*	20.72	Grain width, days to maturity, seed maturation, brown rice shape, average grain weight	(Yoo et al., 2006)

### Candidate gene prediction and *cis*-regulatory analysis of *qZn_5.2_*

**Figure 3:**
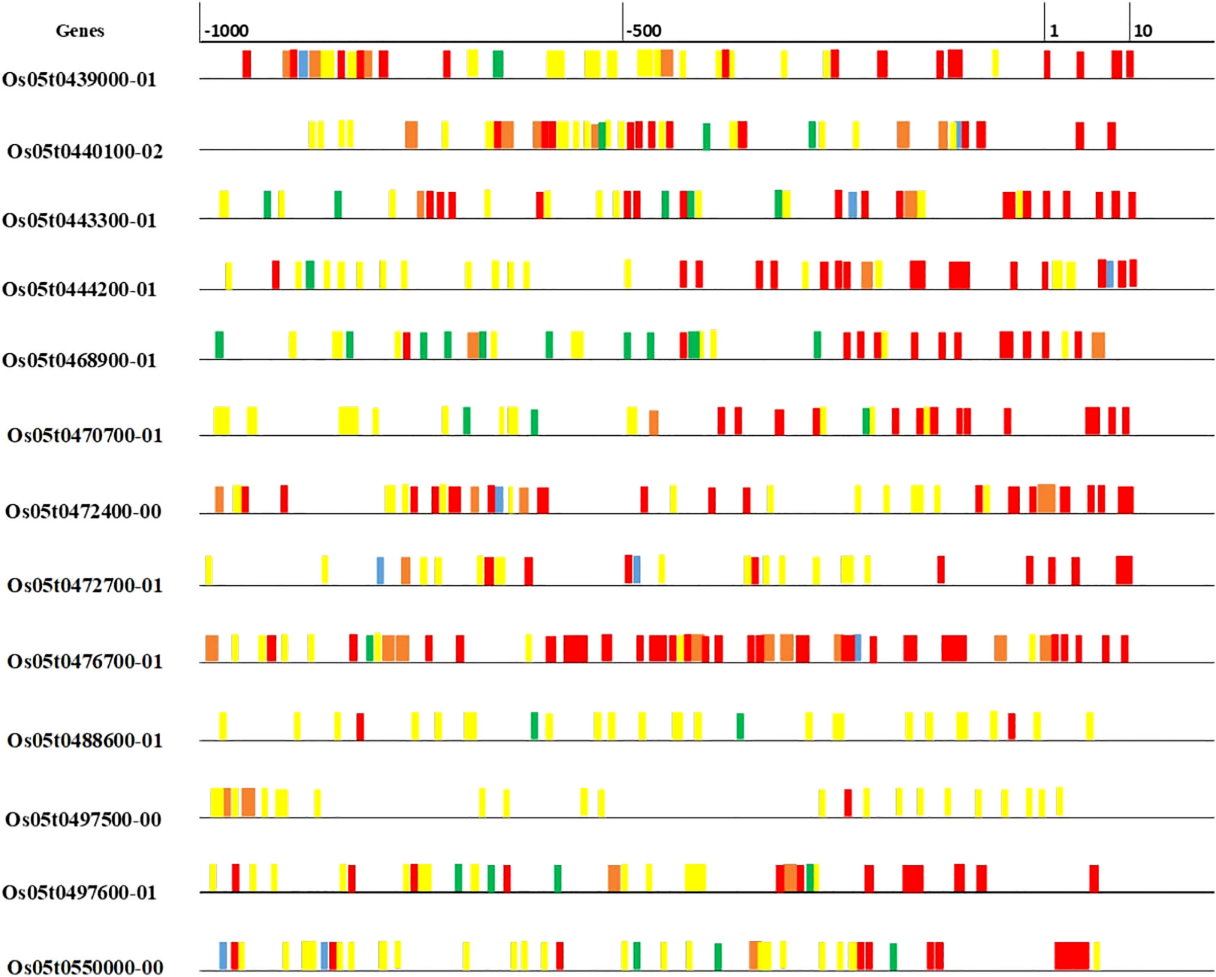
Cis-regulatory elements in the upstream region (500 Kb) of predicted genes for grain Zn. Various elements are associated with physiological functions such as submergence tolerance (blue), merismatic (orange), disease resistance (yellow), light regulation (green), amylase synthesis (red) were indicated using different colors.

The details of 20 candidate genes shortlisted from *qZn_5.2_* are presented in **Table 4**. Most of the candidate genes were either Zn finger or metal cation transporters. Four putative candidate genes identified within or nearest the QTL Viz: *LOC_OS05G39540 (OsZIP9), LOC_OS05G39560 (OsZIP5), LOC_OS05G40490* and LOC*_OS05G41790*. These genes are differentially expressed in roots, leaves, stems, flowers, and meristems (**Figure S4**). *OsZIP5* was highly expressed in stem, internode and seeds, and moderately expressed in roots; *OsZIP9* highly expressed in roots differentiation zone, and in inflorescence. *LOC_OS05G40490* had minimum expression in leaves during drought stress, but *LOC_OS05G41790* has high expression in seeds and had moderate expression in leaves under well-watered and drought conditions (**Figure S4**). The *in-silico* gene prediction analyses of all these genes using the 3k genome identified 75 non-synonymous SNPs, 23 transversions, 34 transitions, and 18 deletions. Out of 20, 15 genes have non-synonymous SNPs found in their promoter and coding sequences (**Table S3**). The highest number of transitions (A/C, A/T, or G/C) was identified for *OsZIP9* and *LOC_OS05g40490*, while *OsZIP9* had the highest number of transversions (A/G or T/G*). LOC_OS05g40490* had the highest number of deletions. The 15 predicted genes that show non-synonymous SNPs were further investigated for *cis-*regulatory elements in their promoter regions using PlantPAN. A large number of *cis*-regulatory elements were detected and they were mainly associated with important physiological processes involved in submergence tolerance, light-regulation, meristematic tissue activities, and disease resistance (**Figure 3**).

**Table 4:** Candidate genes for grain Zn concentration underlying the major QTL (*qZn_5.2_*).

Gene Name	Locus Name	Gene Product Name	Position	Ontology Classification	References
	*LOC_OS05G36090*	Zn finger DHHC domain-containing protein, putative, expressed	21.39	signal transduction, multicellular organism development; receptor activity; binding plasma membrane	(http://rice.uga.edu/cgi-bin/ORF_infopage.cgi?orf=LOC_Os05g36090)
	*LOC_OS05G36310*	Zn finger C3HC4 type containing protein, expressed	21.51	Zn ion binding; metal ion binding; protein binding	(Wu et al., 2014; http://rice.uga.edu/cgi-bin/ORF_infopage.cgi?orf=LOC_Os05g36310)
	*LOC_OS05G37120*	sec23/sec24 Zn finger family protein	21.7	Zn ion binding; intracellular protein transport	(http://rice.uga.edu/cgi-bin/ORF_infopage.cgi?orf=LOC_Os05g37120)
	*LOC_OS50G37190*	C2H2 zinc finger protein, expressed	21.73	metal ion binding; nucleic acid binding	(Han et al., 2020; http://rice.uga.edu/cgi-bin/ORF_infopage.cgi?orf=LOC_Os05g37190)
	*LOC_OS50G37900*	Zn finger C3HC4 type containing protein, expressed	22.21	Zn binding	(Wu et al., 2014; http://rice.uga.edu/cgi-bin/ORF_infopage.cgi?orf=LOC_Os05g37900)
	*LOC_OS05G38360*	DHHC Zn finger domain-containing protein	22.49	binding; biological processes	(Xiang et al., 2010; http://rice.uga.edu/cgi-bin/ORF_infopage.cgi?orf=LOC_Os05g38360)
*ZOS20*	*LOC_OS50G38620*	ZOS20-10-C2H2 Zn finger protein	22.65	biosynthetic processes; nucleobase, nucleoside, nucleotide, and nucleic acid metabolic process; binding; intracellular; sequence-specific DNA binding transcription factor	(Fedotova et al., 2017; http://rice.uga.edu/cgi-bin/ORF_infopage.cgi?orf=LOC_Os05g38620)
	*LOC_OS05G39260*	Zn finger, C3HC4 type domain-containing protein	23.02	Zn ion binding; metal ion binding	(http://rice.uga.edu/cgi-bin/ORF_infopage.cgi?orf=LOC_Os05g39260)
	*LOC_OS05G39380*	Zn finger, C3HC4 type domain-containing protein	23.1	Zn ion binding; metal ion binding; protein binding	(http://rice.uga.edu/cgi-bin/ORF_infopage.cgi?orf=LOC_Os05g39380)
*OsZIP9*	*LOC_OS05G39540*	Metal cation transporter	23.21	transmembrane transport; metal ion transport; Zn ion transmembrane transport; metal ion transmembrane transport; an integral component of membrane	(Yang et al., 2020)
*OsZIP5*	*LOC_OS05G39560*	Metal cation transporter	23.22	transmembrane transport; metal ion transport; Zn ion transmembrane transport; metal ion transmembrane transport; an integral component of membrane	(Lee et al., 2010)
	*LOC_OS05G40490*	Zn knuckle domain-containing protein	23.78	Zn binding	(http://rice.uga.edu/cgi-bin/ORF_infopage.cgi?orf=LOC_Os05g40490)
	*LOC_OS05G41520*	Zn finger, C3HC4 type domain-containing protein	24.28	Zn binding	(http://rice.uga.edu/cgi-bin/ORF_infopage.cgi?orf=LOC_Os05g41520)
*ZOS5*	*LOC_OS05G41530*	ZOS5-11-C2H2 Zn finger protein	24.31	Zn binding	(Malik et al., 2022; http://rice.uga.edu/cgi-bin/ORF_infopage.cgi?orf=LOC_Os05g41530)
	*LOC_OS05G41790*	Zn finger C-x8-C-x5-C-x3-H type family protein	24.47	Zn ion binding; metal ion binding; nucleic acid binding; positive regulation of vernalization response	(Yang et al., 2018; http://rice.uga.edu/cgi-bin/ORF_infopage.cgi?orf=LOC_OS05G41790)
*OsAIR1*	*LOC_OS05G41795*	Zn finger C3HC4 type containing protein, expressed	24.48	protein methylation; protein methyltransferase activity; Zn ion binding; metal ion binding; protein ion binding	(http://rice.uga.edu/cgi-bin/ORF_infopage.cgi?orf=LOC_Os05g41795)
	*LOC_OS05G44400*	GATA Zn finger domain-containing protein	25.83	biosynthetic process; nucleotide, nucleobase, nucleoside, and nucleic acid metabolic process; response to an abiotic stimulus; sequence-specific DNA binding transcription	(http://rice.uga.edu/cgi-bin/ORF_infopage.cgi?orf=LOC_Os05g44400)
	*LOC_OS05G44550*	Zn finger family protein	25.91	binding	(http://rice.uga.edu/cgi-bin/ORF_infopage.cgi?orf=LOC_Os05g44550)
	*LOC_OS05G45020*	Zn finger/CCCH protein factor	26.17	biosynthetic process; nucleotide, nucleobase, nucleoside, and nucleic acid metabolic process; nucleic binding; sequence-specific DNA binding transcription factor activity	(http://rice.uga.edu/cgi-bin/ORF_infopage.cgi?orf=LOC_Os05g41795)
	*LOC_OS05G47670*	Zn finger, C3HC4 type domain-containing protein	27.32	Zn ion binding; metal ion binding; protein binding	(http://rice.uga.edu/cgi-bin/ORF_infopage.cgi?orf=LOC_Os05g47670)

QTL, Quantitative Trait Loci; Chr, Chromosome; Mb, Megabase pairs.

## Discussion

Breeding for improved nutrition has been prioritized in rice and other major staple food crops (Bouis and Saltzman, 2017; Gaikwad et al., 2020). There have been significant efforts over the last one decade to breed for high Fe, Zn and Vitamin A enriched crop varieties, and to deploy them on a large scale to create a health impact (Mwanga et al., 2021; Swamy et al., 2021a; Biswas et al., 2021). In rice, Zn biofortification breeding has been taken up on a large scale by IRRI and its partners leading to release of several high Zn rice varieties in Asia and Africa (Swamy et al., 2016; Tsakirpaloglou et al., 2019; Calayugan et al., 2021). Recently, Zn mainstreaming breeding has been initiated to incorporate Zn as a must trait in all the future rice varieties (Stangoulis and Knez, 2022). Identification of diverse donors, QTLs, genes, and a better understanding of molecular basis of grain Zn concentration, agronomic and yield traits are essential for the efficient Zn biofortification of rice (Swamy et al., 2016, Swamy et al., 2018; Calayugan et al., 2020; Rakotondramana et al., 2022)

We used high Zn *aus* accession Kaliboro (IRGC 77201-1) to develop four RILs mapping populations. It has acceptable yield potential (~4900 kg/ha) and twice the amount of grain Zn (~40 ppm) in comparison to popular rice varieties (14-16ppm). In addition to that *aus* accessions are also genetically diverse and adaptable to a wide range of environmental conditions (Redoña and Mackill, 1996; Xu et al., 2006; Jagadish et al., 2008; Norton et al., 2010; Henry et al., 2011; Gamuyao et al., 2012; Al-Shugeairy et al., 2014). All the four recipient parents were high Zn breeding lines (12-19 ppm) developed at IRRI.

A wide range of variation for GY, Zn, Fe, and other major agronomic traits was observed in all the populations. Most of the traits exhibited normal distribution indicating the complex genetic basis of these traits (**Table 1** and **Figures S1, S2**). Broad-sense heritability (H^2^) was high for DF, PH, TGW, and Zn across populations (**Table 1**), which permits effective phenotypic selection for their improvement. These results are in consonance with earlier reports on traits distributions and heritability (Widodo et al., 2010; Du et al., 2013; Zhang et al., 2014; Swamy et al., 2016; Chang et al., 2018; Swamy et al., 2018). Consistent positive correlations were observed between Fe and Zn (**Figure 1**) indicating the possibility of simultaneous improvement (Swamy et al., 2016). However, concomitant breeding for high Zn and Fe with high GY will be challenging due to their strong negative correlations. Hence, appropriate breeding strategies would be essential for the successful Zn biofortification (Welch, 2004; Chang et al., 2018). One approach is to use high Zn donors with acceptable GY potential coupled with rapid generation advancement, speed breeding and genomic selection (Swamy et al., 2016; Calayugan et al., 2021; Swamy et al., 2021a; Yang et al., 2021). Moreover, breeding materials or populations that exhibit weak or no correlation between GY and Zn and transgressive seggregants that possess both high GY and Zn increases the chances of successful biofortification breeding (**Figures 1**, **S1**).

### MC-QTL analysis detected major QTLs and epistasis

Genetic analyses using multi-parent derived populations or combined analysis of multiple biparental populations that share common parentage help to dissect major effect and stable QTLs, which can work across genetic backgrounds without any epistatic or genetic background effects (Specht et al., 2001; Jannink and Jansen, 2001; Blanc et al., 2006; Doust et al., 2014). We carried out MC-QTL analyses for 11 traits using a set of 495 SNPs distributed across 12 chromosomes (**Figure S5**). A high rate of SNPs polymorphism among parents was expected as Kaliboro (*aus*) and IR14M141, IR14M110, IR14M125, and IR95044:8-B-5-22-19-GBS (*indica*) are from genetically-distinct subgroups (Garis et al., 2005; Chen et al., 2014; Tang et al., 2016). The MC-QTL analysis detected more QTLs but effects were smaller compared to QTLs discovered by Inclusive Composite Interval Mapping (ICIM) (**Tables 2** and **S4**). For instance, MC-QTL was able to detect 14 QTLs for Fe whereas only 2 QTLs were identified by ICIM. Some of the important QTLs detected using MC-QTL analysis viz: *qZn_5.2_, qFe_7.1_, qGY_10.1_, qDF_7.1_, qPH_1.1_, qNT_4.1_, qPT_4.1_, qPL_1.2_, qTGW_5.1_, qGL_3.1_*, and *qGW_6.1_*, which can be used in rice MAB and Genomic Selection (GS). Interestingly, most of the positive alleles for high GY and early DF were contributed by high yielding (IR14M110) and early maturing parents (IR95044:8-B-5-22-19-GBS) respectively. High-grain yield and early-maturity are two most desirable traits for rice varietal development (Zhao et al., 2011; MacKill and Khush, 2018; Li et al., 2019). Similarly, QTLs for PL and GW were contributed by Kaliboro, while TGW and GL were derived from IR14M141 (**Table 2**). Thus, it is evident that Kaliboro has contributed many positive alleles for yield and yield related traits and for improved grain Zn concentration. These favorable alleles can be pooled through genomics assisted breeding to develop rice varieties (Blanc et al., 2006; Grenier et al., 2015; Varshney et al., 2021; Xu et al., 2021; Reyes et al., 2022).

The QTLs of correlated traits (positive or negative) tend to cluster together on a chromosomal region (Zhang et al., 2019; Yu et al., 2021). The GY, TGW, GL, and GW were highly correlated, and their respective QTLs co-located on chromosome 5. All the QTLs for PT and NT, and Fe and Zn were co-located (**Figures 1**, **2**). However, these co-locations may be due to pleiotropy or linkage or shared regulatory genes (Collard and MacKill, 2007; Du et al., 2013; Swamy et al., 2016; Descalsota et al., 2018). Positively correlated traits and their co-located QTLs can be readily mobilized into the breeding programs, while negative linkages must be removed by pre breeding, phenotypic selections, and fine mapping for their deployment in the rice breeding (Du et al., 2013; Swamy et al., 2016; Descalsota et al., 2018; Descalsota-Empleo et al., 2019a; Pradhan et al., 2020).

A notable result is that a major QTL for grain Zn (*qZn_5.2_*) is consistently identified in all the populations and also in the combined population analysis. It had a R^2^ of 13% in MC-QTL analysis and went up to 19.75% in ICIM (**Table S4**), with an additive effect ranging from 0.44 to 2.10 ppm (**Tables 2** and **S4**), and had a narrow genetic interval of 600Kb. It is also devoid of any epistatic interaction or background effects, making it one of the potential candidate loci for MAB or GS to improve grain Zn concentration. This locus has been frequently reported from *aus* derived populations. Several studies have also identified similar genomic regions (Lu et al., 2008; Garcia-Oliveira et al., 2009; Zhang et al., 2014; Descalsota-Empleo et al., 2019b; Islam et al., 2020).

We observed very minimal epistasis among the QTLs. The PH (*qPH_6.1_*) interacted with GY (*qGY_6.1_*) and Zn (*qZn_6.1_*), but their R^2^ (<5%) and additive effects (0.12-1.25 cm, 9-282 kg/ha, 0.13-1.0 ppm, respectively) were low to make any significant changes in their phenotypic expression (**Table S2**). There were reports on genetic interactions between Zn and PH that affected their phenotypic performance, and also genetic background altering the epistasis effects leading to variable trait expression (Jiang et al., 2008; Lekklar et al., 2019). Therefore, epistatic QTLs and the influence of genetic backgrounds on major QTLs should be verified before their use in the breeding programs (Xu et al., 2015; Islam et al., 2019; Lekklar et al., 2019).

### Candidate genes co-located with major QTLs

A number of candidate genes that co-localized with QTLs were identified for different traits such as *HUA2* (DF), *OsPdk1* (PL), *OS01G0899425* (PH)*, OS04G0350700* (PT), *GSK2* (TGW), *OS10G0325400* (YLD)*, GS3* (GL), and *GSK4* (GW) (**Table 3**). These genes have proven biological functions directly or indirectly related to the traits but also involved in the important physiological processes related to stress tolerance (Chen and Meyerowitz, 1999; Fan et al., 2006; Jan and Komatsu, 2006; Yoo et al., 2006; Wei et al., 2012; Hu et al., 2015; Jan and Komatsu, 2006; Li et al., 2016; Yin et al., 2019; Sevanthi et al., 2021). Previously reported QTLs and genes for yield and yield related traits and grain micronutrients match with our results (Anuradha et al., 2012; Du et al., 2013; Descalsota et al., 2018; Zhang et al., 2019; Pradhan et al., 2020). Some of these genes are potential candidates for functional validation and for introgression through MAB.

The *qZn_5.2_* was also enriched with Zn/Metal homeostasis genes, especially Zn-finger proteins (**Table 3**). They play an important role in Zn uptake, transport and loading into the grains, and their expression provides abiotic stress tolerance under diverse climatic conditions (Goel et al., 2011; Zhang et al., 2014; Hara et al., 2017; Papierniak et al., 2018; Lekklar et al., 2019). It is noteworthy that these genes were also enriched with *cis*-regulatory elements in their promoter regions, and actively regulates physiological processes involved in metal homeostasis, submergence tolerance, meristematic tissue activities, light-regulation and disease resistance (Pandey et al., 2015; Biłas et al., 2020) (**Table 4** and **Figure 3**).

Functional polymorphisms within genes play an important role in phenotypic diversity (Srivastava et al., 2014). We report that 15 of the 20 genes shortlisted from *qZn_5.2_* had functional polymorphic SNPs; notable ones are *OsZIP9*, *OsZIP5*, and *LOC_OS05G40490*. These transcription factors regulate expression of Zn homeostasis genes such as *OsNAS1*, *OsNAS2*, and *OsNAS3*, therefore play a role in metal transport, partitioning and loading into grains (Guerinot, 2000; Chandel et al., 2010). There are previous reports showing upregulation of *OsZIP5* in roots and flag leaves, and *OsZIP9* in roots of rice (Banerjee and Chandel, 2011; Chadha-Mohanty et al., 2015), but they are homologs and complement in phytosiderophore-mediated Zn uptake (Lee et al., 2010). Recent haplotype analysis of *OsZIP5* and *OsZIP9* using a 3K rice panel revealed that superior haplotypes for these two genes were in high frequency in *aus* and *indica* sub groups (Gaurav et al., in press). On the other hand, there is less information on *LOC_OS05G40490*, a Zn knuckle domain-containing protein gene involved in metal binding. Some of these genes can be pyramided using gene specific markers, explored for superior haplotypes, and can be used to develop high Zn transgenics or genome edited rice lines.

## Conclusion

We carried out MC-QTL and ICIM analysis for agronomic, yield and grain micronutrient traits using connected rice populations. In all 156 MC-QTLs, 26 QTL co-locations and two epistatic interactions were detected. The donor parent, Kaliboro, contributed many positive alleles. A major QTL (*qZn_5.2_*) for grain Zn concentration has been detected on chromosome 5 that accounted for 13% of R^2^. There were 20 genes within *qZn_5.2_* that are involved in metal binding, uptake, transport, partitioning and loading in different tissues. Three primary putative genes, *OsZIP5*, *OsZIP9*, and *LOC_OS05G40490*, along with 12 other genes showed functional SNP polymorphisms indicating their role in metal homeostasis. These genes were also enriched with *cis*-regulatory elements that regulate mineral uptake, growth and development, and tolerance to biotic and abiotic stresses etc. All the major effect QTLs can be readily mobilized into breeding programs through genomics assisted breeding. Promising candidate genes can be characterized to understand their functional roles. Over all our results provide insights into molecular bases of Zn homeostasis, and identified major QTLs/genes useful for Zn biofotification of rice.

## Data availability statement

The phenotypic and genotypic data used in the analyses are provided as supplementary data in this article.

## Author contributions

AP and BS designed the paper. AP led the conduct of the experiment and wrote the whole manuscript. CN, GD-E, MC, and ZS analyzed and gathered some genotypic and phenotypic data. AA and MI-A assisted in the establishment of field experiments and collection of phenotypic data. JH, PS, TB, AL, RM, KM and BS thoroughly edited and contributed significantly to the improvement of the manuscript. All authors contributed to the article and approved the submitted version.
