# Supplementary material for: Molecular dissection of connected rice populations revealed important genomic regions for agronomic and biofortification traits
Source: Front Plant Sci. 2023 Mar 22;14:1157507. doi: 10.3389/fpls.2023.1157507 (PMC10073715; doi:10.3389/fpls.2023.1157507)
Supplement: Supplementary file 1 [file DataSheet_1.docx]

**Supplementary Tables**

**Table S1.** Details of the RILs Populations used for the MC-QTL analysis.

| **Population** | **Parents** | **Population Size** | **Type of Population** |
| --- | --- | --- | --- |
| Pop1 | IR14M141 (P1) x Kaliboro::IRGC 77201-1 (P2) | 360 | RIL |
| Pop2 | IR14M110 (P3) x Kaliboro::IRGC 77201-1 (P2) | 120 | RIL |
| Pop3 | IR14M125 (P4) x Kaliboro::IRGC 77201-1 (P2) | 210 | RIL |
| Pop4 | IR95044:8-B-5-22-19-GBS (P5) x Kaliboro::IRGC 77201-1 (P2) | 205 | RIL |

**Table S2.** Epistatic effects identified between Zn and PH QTL.

| **Chromosome** | **QTL position** | **QTL with epistatic effect** | | **% R^2^ Value** |
| --- | --- | --- | --- | --- |
|  |  | **Chromosome** | **Position** |  |
| 5 | 22.03 | 6 | 8.72 | **5** |
| 6 | 8.72 | 6 | 24.24 | **5.4** |

**Table S3.** SNP polymorpshisms within the candidate genes.

| **Gene Name** | **Locus Name** | **Chromosome** | **Transition** | **Transversion** | **Deletion** |
| --- | --- | --- | --- | --- | --- |
|  | *LOC_OS05g36310* | 5 |  | 1 |  |
|  | *LOC_OS05g36910* | 5 | 1 | 2 |  |
|  | *LOC_OS05g37120* | 5 | 1 | 1 |  |
|  | *LOC_OS053g7190* | 5 | 4 |  |  |
|  | *LOC_OS05g39260* | 5 | 1 | 1 |  |
|  | *LOC_OS05g39380* | 5 | 2 | 2 |  |
| *OsZIP9* | *LOC_OS05g39540* | 5 | 8 | 5 | 2 |
| *OsZIP5* | *LOC_OS05g39560* | 5 | 1 | 3 |  |
|  | *LOC_OS05g39930* | 5 | 3 |  | 2 |
|  | *LOC_OS05g40490* | 5 | 8 | 3 | 14 |
|  | *LOC_OS05g40960* | 5 | 1 |  |  |
|  | *LOC_OS05g41790* | 5 | 1 | 2 |  |
| *OsAIR1* | *LOC_OS05g41795* | 5 |  | 1 |  |
|  | *LOC_OS05g44550* | 5 | 1 |  |  |
|  | *LOC_OS05g47670* | 5 | 2 | 2 |  |

**Table S4.** QTL identified for Zn, Fe, GY, and yield-related traits using ICIM in four individual RIL populations.

| **SN** |  | **Trait** | **QTL** | **Chr** | **Marker Interval** | **Position (Mbp)** | **LOD** | **PVE (%)** | **Additive** | **Population** |
| --- | --- | --- | --- | --- | --- | --- | --- | --- | --- | --- |
| 1 |  | Zn | *qZn_2.1_* | 2 | 2035959-2096898 | 20.74-22.62 | 11.24 | 21.49 | -3.03 | POP1 |
| 2 |  |  | *qZn_2.2_* | 2 | 2096898-SNP-2.24170823. | 22.62-24.17 | 6.25 | 17.94 | -1.97 | POP1 |
| 3 |  |  | *qZn_4.1_* | 4 | id4003793-id4010238 | 12.34-30.39 | 6.08 | 11.91 | 1.68 | POP3 |
| 4 |  |  | *qZn_5.1_* | 5 | 4928297-4931423 | 4.12-4.23 | 5.60 | 11.52 | -0.82 | POP4 |
| 5 |  |  | *qZn_5.1_* | 5 | 5337933-5622212 | 14.66-22.93 | 3.04 | 10.18 | -1.08 | POP1 |
| 6 |  |  | *qZn_5.2_* | 5 | 5579383-5762361 | 26.16-28.13 | 10.71 | 16.00 | -1.66 | POP1 |
| 7 |  |  | *qZn_5.1_* | 5 | 5579383-5711540 | 27.08-28.13 | 3.61 | 6.94 | -1.48 | POP2 |
| 8 |  |  | *qZn_5.1_* | 5 | 5711540-5726844 | 26.16-26.76 | 11.31 | 19.75 | -2.10 | POP3 |
| 9 |  |  | *qZn_6.1_* | 6 | id6009910-id6010136 | 17.75-18.18 | 9.17 | 16.83 | -2.30 | POP1 |
| 10 |  |  | *qZn_6.1_* | 6 | 6877746-6906770 | 28.33-29.33 | 5.78 | 7.44 | -1.24 | POP2 |
| 11 |  |  | *qZn_7.1_* | 7 | 7809062-7824699 | 23.35-23.99 | 4.88 | 7.90 | -1.60 | POP1 |
| 12 |  |  | *qZn_8.1_* | 8 | 8331918-8372006 | 9.28-10.10 | 4.39 | 11.39 | 1.32 | POP1 |
| 13 |  |  | *qZn_8.2_* | 8 | 8969762-9016485 | 25.30-26.95 | 3.32 | 8.58 | -1.45 | POP1 |
| 14 |  |  | *qZn_10.1_* | 10 | 10651050-10678891 | 17.15-18.02 | 6.35 | 13.01 | 0.88 | POP4 |
| 15 |  |  | *qZn_12.1_* | 12 | 12641039-12853828 | 15.62-20.73 | 11.39 | 20.67 | -2.17 | POP3 |
| 16 |  | Fe | *qFe_3.1_* | 3 | SNP-3.18196421.-SNP-3.9896907. | 9.89-18.20 | 5.93 | 13.80 | -0.17 | POP3 |
| 17 |  |  | *qFe_9.1_* | 9 | 9563291-9565186 | 12.15-12.21 | 7.13 | 26.24 | 0.49 | POP1 |
| 18 |  | GY | *qYLD_1.1_* | 1 | 1122670- SNP-1.34676800 | 34.35-34.68 | 3.93 | 5.85 | 292.14 | POP2 |
| 19 |  |  | *qYLD_2.1_* | 2 | 1543608-SNP-2.8455563. | 6.47-8.45 | 5.52 | 15.85 | -587.52 | POP1 |
| 20 |  |  | *qYLD_2.2_* | 2 | 2035959-2096898 | 20.74-22.62 | 6.33 | 15.67 | 610.51 | POP1 |
| 21 |  |  | *qYLD_3.1_* | 3 | id3010896 -id3010971 | 24.73-25.14 | 4.31 | 10.52 | -344.48 | POP1 |
| 22 |  |  | *qYLD_7.1_* | 7 | 7745053-rd7002196 | 21.31-21.77 | 6.57 | 27.64 | 758.20 | POP1 |
| 23 |  | DF | *qDF_2.1_* | 2 | 1543608 -SNP-2.8455563. | 6.47-8.45 | 8.35 | 8.10 | 3.49 | POP1 |
| 24 |  |  | *qDF_4.1_* | 4 | SNP-4.203305-597102 | 0.20-0.60 | 9.97 | 9.94 | -2.64 | POP1 |
| 25 |  |  | *qDF_4.2_* | 4 | 3619998-3623485 | 1.04-11.36 | 19.23 | 24.26 | 3.82 | POP1 |
| 26 |  |  | *qDF_5.1_* | 5 | SNP-5.24497575.-id5013100 | 24.56-27.08 | 5.63 | 5.19 | 2.31 | POP1 |
| 27 |  |  | *qDF_7.1_* | 7 | 7006027-ud7000154 | 1.76-2.03 | 6.07 | 5.54 | 1.93 | POP1 |
| 28 |  |  | *qDF_7.2_* | 7 | SNP-7.22188645.-7780169 | 22.18-22.38 | 14.09 | 15.44 | 3.54 | POP1 |
| 29 |  |  | *qDF_7.1_* | 7 | id7005418- id7005665 | 27.55-28.25 | 5.37 | 6.92 | 2.61 | POP2 |
| 30 |  | PH | *qPH_1.1_* | 1 | SNP-1.35192029.- id1022407 | 35.19-35.54 | 3.99 | 5.18 | -5.15 | POP2 |
| 31 |  |  | *qPH_1.1_* | 1 | SNP-1.38961387.-SNP-1.39020597. | 38.96-39.02 | 19.54 | 37.29 | -10.43 | POP3 |
| 32 |  |  | *qPH_1.2_* | 1 | 1250974-id1024973 | 39.08-39.37 | 14.28 | 29.54 | -8.56 | POP4 |
| 33 |  |  | *qPH_3.1_* | 3 | id3004023-SNP-2666586 | 5.92-7.97 | 4.14 | 8.02 | 5.84 | POP1 |
| 34 |  |  | *qPH_3.2_* | 3 | id3016444 -3495083 | 33.69-33.71 | 3.63 | 8.67 | -7.36 | POP1 |
| 35 |  |  | *qPH_6.1_* | 6 | 6048208-6113593 | 7.50-9.40 | 7.36 | 11.71 | -5.75 | POP3 |
| 36 |  |  | *qPH_7.1_* | 7 | 7066806-7066952 | 4.22-4.23 | 5.22 | 6.41 | -0.18 | POP3 |
| 37 |  |  | *qPH_7.1_* | 7 | SNP-7.22188645.-7780169 | 22.18-22.38 | 9.31 | 23.75 | 12.02 | POP1 |
| 38 |  |  | *qPH_7.1_* | 7 | 7810320- SNP-7_26972908 | 23.39-26.97 | 3.83 | 6.01 | 5.50 | POP2 |
| 39 |  |  | *qPH_9.1_* | 9 | 9569595-id9006988 | 12.32-19.48 | 6.04 | 11.70 | -6.82 | POP1 |
| 40 |  | NT | *qNT_4.1_* | 4 | 3647133-id4001482 | 1.71-3.63 | 5.94 | 10.81 | -0.48 | POP4 |
| 41 |  | NP | *qNP_4.1_* | 4 | 3647133-id4001482 | 1.71-3.63 | 5.74 | 10.42 | -0.47 | POP4 |
| 42 |  | PL | *qPL_1.1_* | 1 | 1028287- SNP-1.31640214. | 30.99-31.64 | 6.39 | 7.94 | -0.87 | POP2 |
| 43 |  |  | *qPL_1.2_* | 1 | 1250974-id1024973 | 39.08-39.37 | 9.40 | 14.43 | -0.72 | POP4 |
| 44 |  |  | *qPL_6.1_* | 6 | SNP-6.5731592.- id6004481 | 5.73-7 | 3.65 | 5.13 | 0.61 | POP2 |
| 45 |  |  | *qPL_9.1_* | 9 | 9569595-id9006988 | 12.32-19.48 | 4.15 | 16.40 | -1.16 | POP1 |
| 46 |  | TGW | *qTGW_1.1_* | 1 | 882462-id1018601 | 26.20-30.85 | 6.68 | 13.34 | -0.68 | POP4 |
| 47 |  |  | *qTGW_2.1_* | 2 | 2422788-2430179 | 33.51-33.77 | 93.91 | 5.38 | 6.27 | POP3 |
| 48 |  |  | *qTGW_3.1_* | 3 | 2666586-id3010896 | 7.97-24.73 | 5.21 | 17.84 | -0.99 | POP1 |
| 49 |  |  | *qTGW_5.1_* | 5 | 4953842- id5002699 | 4.8-5.29 | 3.84 | 5.16 | 0.67 | POP2 |
| 50 |  |  | *qTGW_6.1_* | 6 | 6737662-6749451 | 23.9-24.33 | 4.36 | 6.20 | -0.72 | POP2 |
| 51 |  |  | *qTGW_6.1_* | 6 | 5901730-id6002884 | 28.08 | 4.05 | 13.65 | 0.88 | POP1 |
| 52 |  |  | *qTGW_6.2_* | 6 | 6906770-6907224 | 29.33-29.34 | 5.92 | 24.31 | -1.11 | POP1 |
| 53 |  |  | *qTGW_7.1_* | 7 | 7066806-7066952 | 4.22-4.23 | 13.82 | 16.23 | -0.86 | POP3 |
| 54 |  |  | *qTGW_7.2_* | 7 | id7004041-7810320 | 23.09-23.39 | 3.63 | 5.04 | 0.71 | POP2 |
| 55 |  |  | *qTGW_12.1_* | 12 | SNP-12.11050627.-SNP-12.13955798. | 11.05-13.96 | 5.30 | 5.61 | 0.50 | POP3 |
| 56 |  | GL | *qGL_1.1_* | 1 | SNP-1.22352640.-SNP-1.24413541. | 22.35-24.41 | 9.88 | 19.39 | -0.30 | POP4 |
| 57 |  |  | *qGL_3.2_* | 3 | 2584928- id3002805 | 4.57-4.76 | 3.43 | 5.23 | 0.14 | POP2 |
| 58 |  |  | *qGL_6.1_* | 6 | 6873320-6877746 | 28.17-28.32 | 16.03 | 43.16 | -0.42 | POP1 |
| 59 |  |  | *qGL_7.1_* | 7 | 7765322-7782475 | 21.89-22.44 | 4.79 | 7.12 | 0.18 | POP2 |
| 60 |  |  | *qGL_8.1_* | 8 | 8307065-id8002954 | 8.70-9.20 | 4.37 | 11.08 | -0.22 | POP1 |
| 61 |  |  | *qGL_12.1_* | 12 | 12016398-12048140 | 0.51-1.75 | 6.46 | 20.10 | 0.18 | POP3 |
| 62 |  | GW | *qGW_1.1_* | 1 | 135989-139967 | 4.16-4.30 | 7.24 | 8.93 | -0.02 | POP1 |
| 63 |  |  | *qGW_2.1_* | 2 | 2096898-SNP-2.24170823 | 22.62-24.17 | 6.35 | 19.74 | -0.12 | POP1 |
| 64 |  |  | *qGW_3.1_* | 3 | id3009824-3113846 | 21.06-21.23 | 3.16 | 5.21 | -0.05 | POP2 |
| 65 |  |  | *qGW_3.1_* | 3 | id3010896-id3010971 | 24.73-25.14 | 5.14 | 15.45 | -0.07 | POP1 |
| 66 |  |  | *qGW_5.1_* | 5 | 4928297-4966908 | 4.12-5.23 | 3.89 | 6.27 | 0.05 | POP2 |
| 67 |  |  | *qGW_5.1_* | 5 | 4953842-4966908 | 4.79-5.23 | 5.83 | 10.05 | 0.12 | POP3 |
| 68 |  |  | *qGW_5.2_* | 5 | 5711540-5726844 | 26.16-26.76 | 5.02 | 6.34 | -0.02 | POP2 |
| 69 |  |  | *qGW_6.1_* | 6 | 6619487-6637446 | 20.98-21.42 | 9.81 | 17.89 | -0.15 | POP3 |
| 70 |  |  | *qGW_11.1_* | 11 | 10919433-ud11000156 | 3.68-3.80 | 3.93 | 11.59 | -0.06 | POP1 |

**Supplementary Figures**


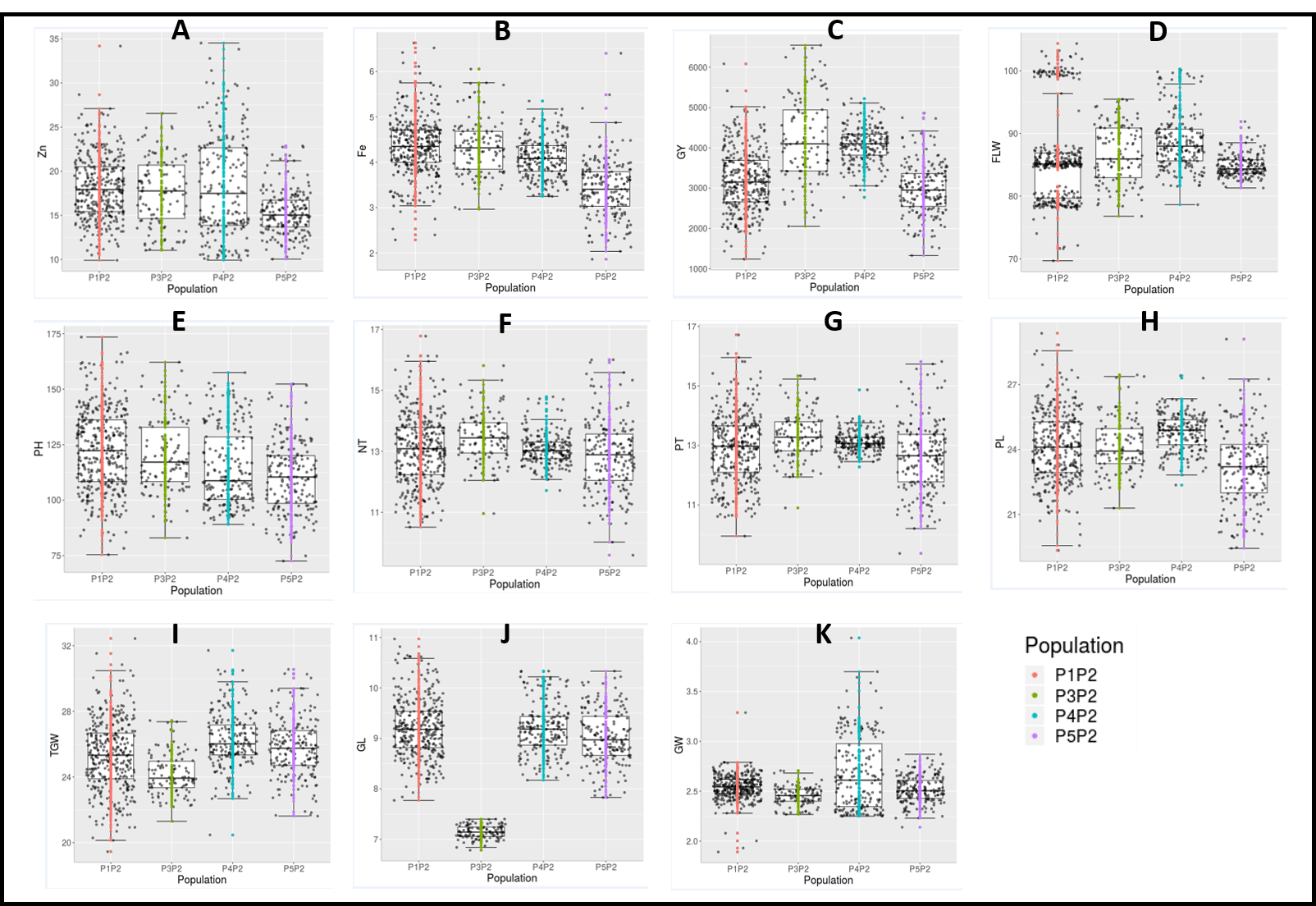


**Figure S1.** Boxplot and scatter plot diagrams with mean and distribution of various traits tested using four connected populations and 11 traits: Zn (A), Fe (B), GY (C), FLW (D), PH (E), NT (F), PT (G), PL (H), TGW (I), GL (J), and GW (K).


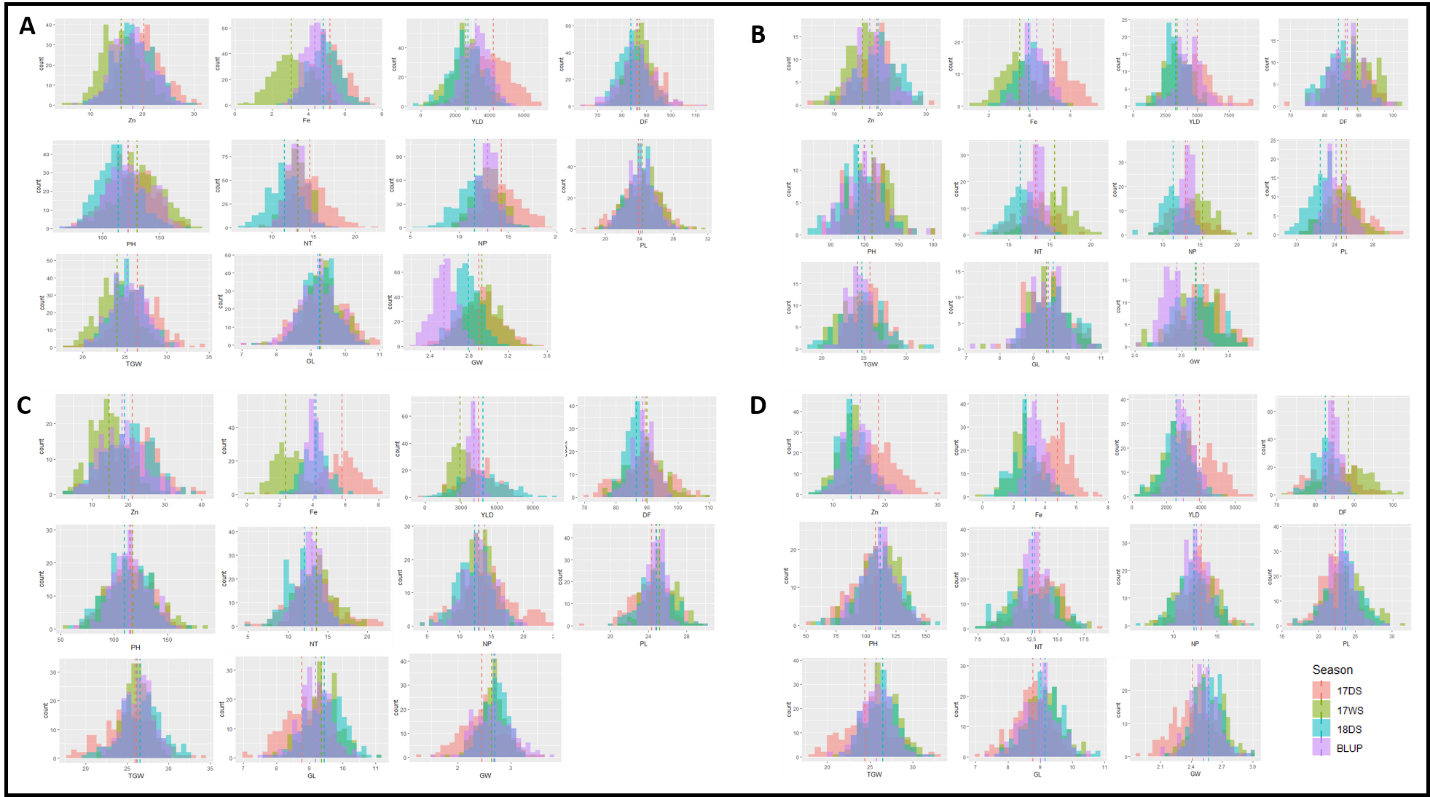


**Figure S2.** Frequency distribution of various traits tested for four connected populations: POP1 (A), POP2 (B), POP3 (C), and POP4 (D) in 2017DS, 2017WS, and 2018DS and combined data using best linear unbiased prediction (BLUP) values.


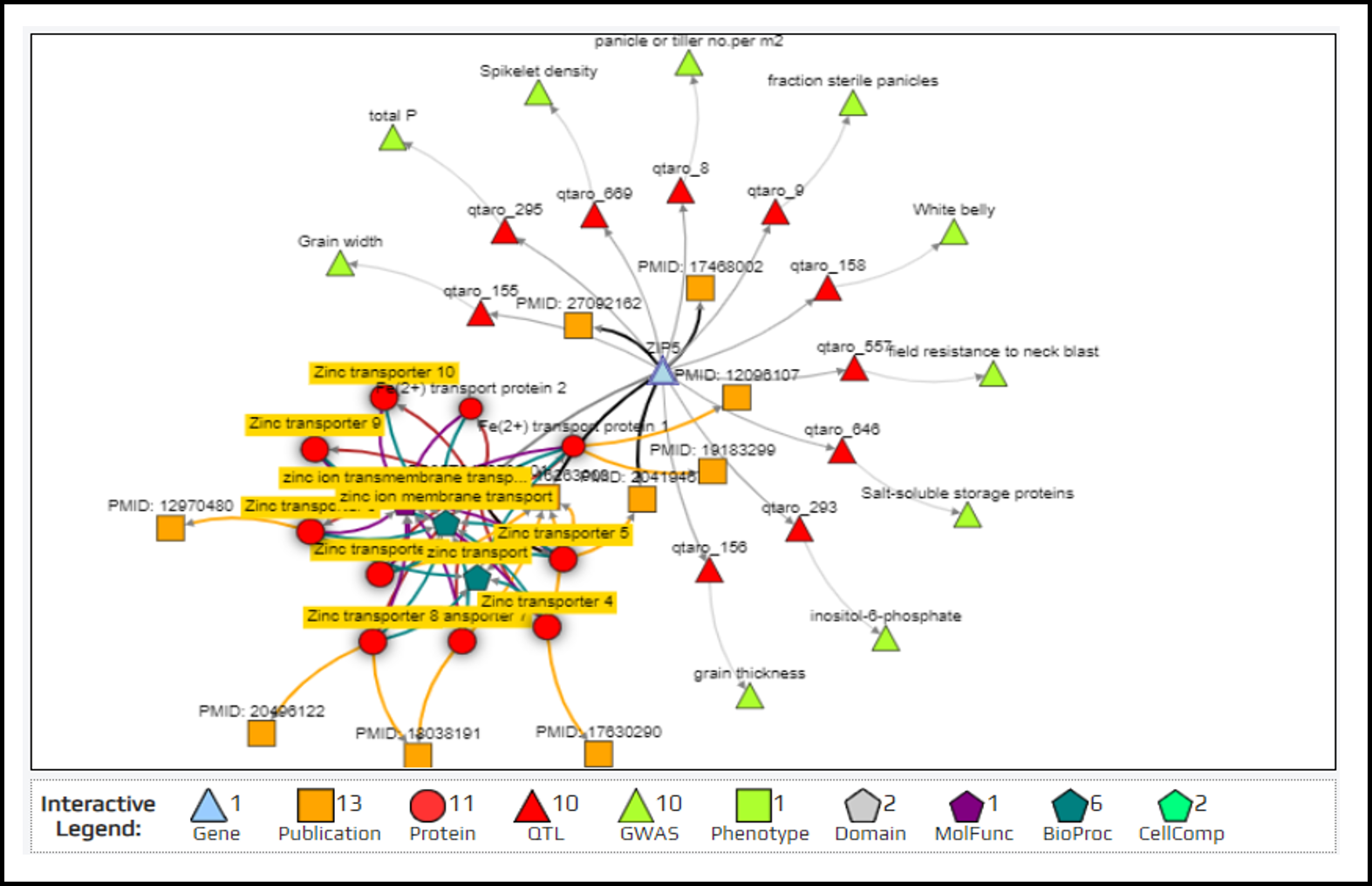


**Figure S3.** Gene network of candidate genes for Zn generated using KnetMiner.


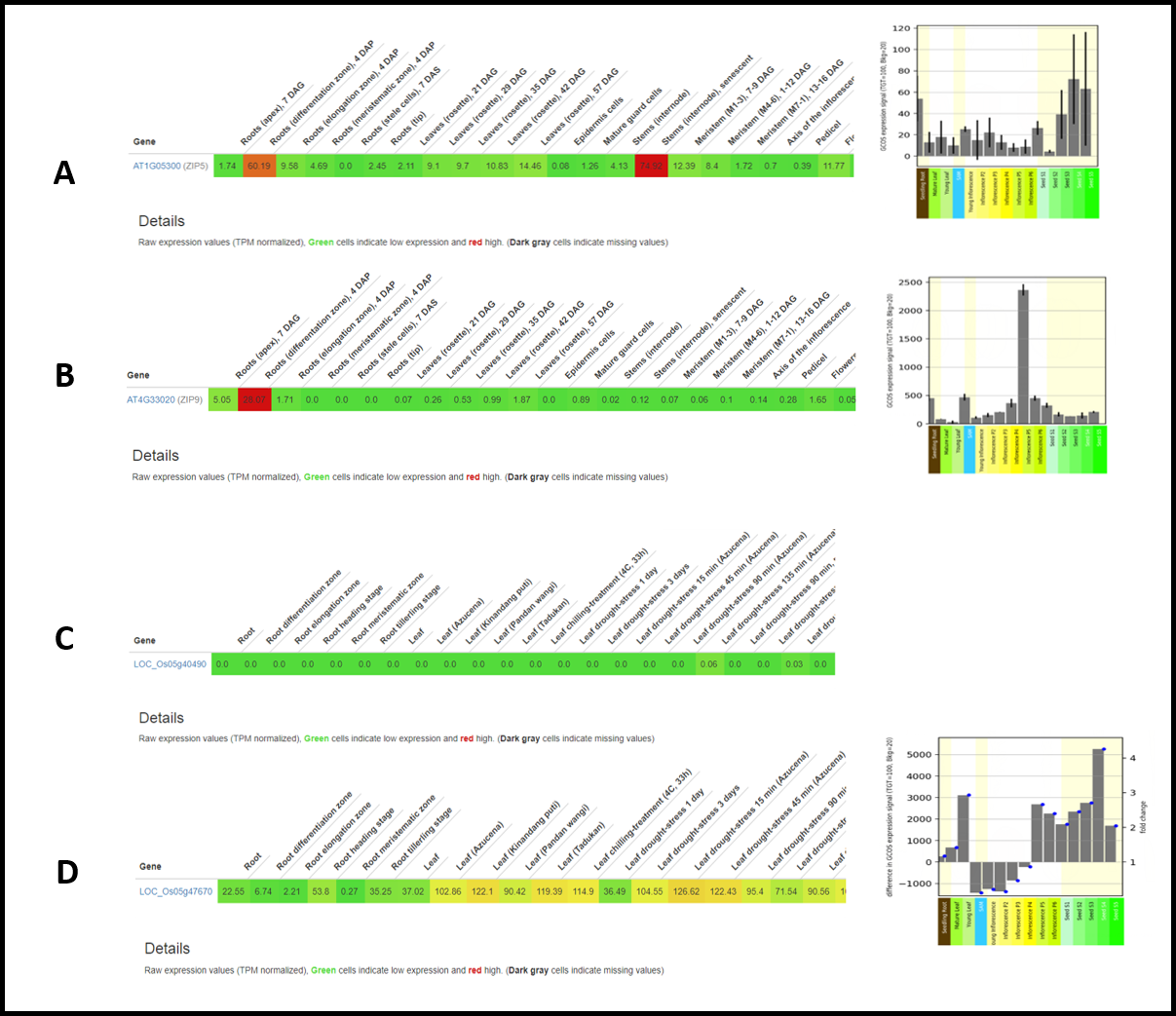


**Figure S4.** Expression of primary candidate genes: *OsZIP5* (A), *OsZIP9* (B*), LOC_Os05g40490* (C), and *LOC_Os05g47670* (D) in various parts of the plants.


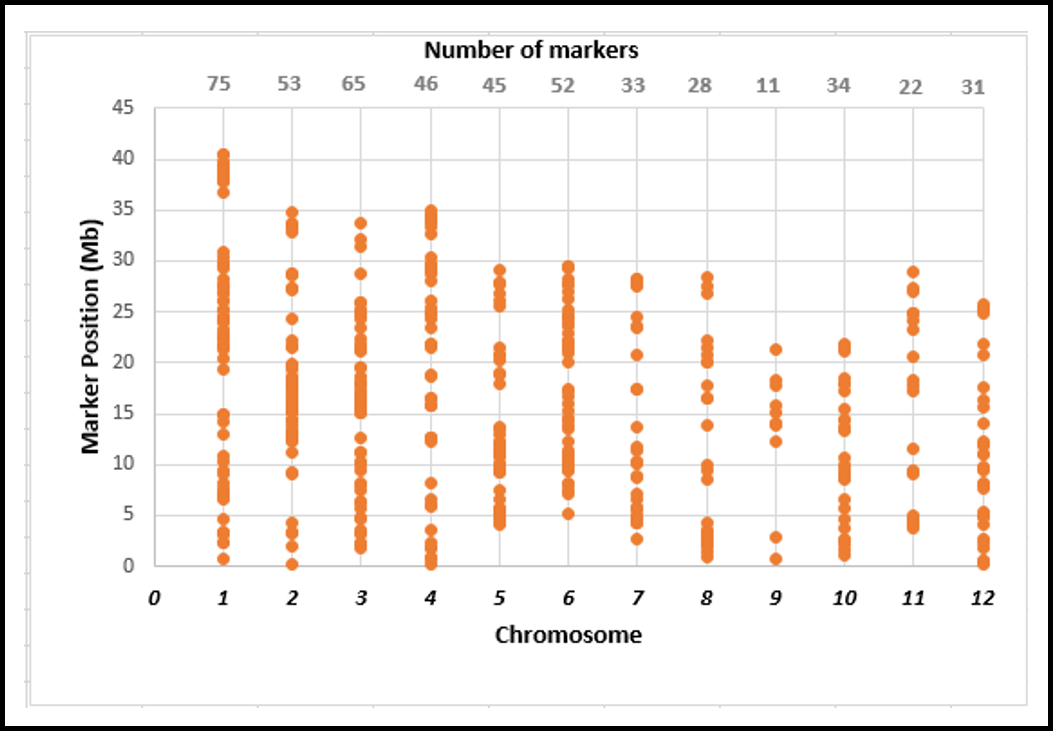


**Figure S5.** Distribution of high-quality SNP markers on 12 chromosomes used in MC-QTL analysis.
